# Development of Versatile Nanoparticle System for Efficient Drug Delivery into Pulmonary Endothelium

**DOI:** 10.1002/advs.78292

**Published:** 2026-10-14

**Authors:** Afzaal N. Mohammed, Zicheng Deng, Arnab Misra, Marcus Orzabal, Ananya Kondapally, Enhong Li, Jonathan Do, Ying‐Wei Lan, Fatemeh Kohram, Hunter Blackburn, Andreas Damianos, Tanya V. Kalin, Vladimir V. Kalinichenko

**Affiliations:** ^1^ Phoenix Children's Research Institute, Department of Child Health University of Arizona College of Medicine‐Phoenix Phoenix USA; ^2^ Division of Neonatology Phoenix Children's Hospital Phoenix USA; ^3^ Center for Cancer and Blood Diseases Phoenix Children's Hospital Phoenix USA

**Keywords:** endothelial cells, FOXF1 transcription factor, nanoparticle drug delivery, neonatal angiogenesis and alveolarization, neonatal lung injury

## Abstract

Drug delivery precisely to the pulmonary vasculature represents a formidable challenge. Pediatric Acute Respiratory Distress Syndrome (PARDS) is an urgent public health concern accounting for high mortality and long‐term morbidity due to severe damage to lung endothelial cells (ECs). Advanced target‐specific therapeutic interventions to protect lung vasculature in PARDS are in dire need. FOXF1 protein is a critical mediator of lung angiogenesis and vascular repair after injury. However, whether FOXF1 is a useful pharmacological target for PARDS is unknown. Herein, we developed a universal nanoparticle‐based Lung Endothelial Drug Delivery (LEDD) system to deliver hydrophobic small‐molecule compounds specifically to lung ECs but not to other cells or organs. Based on single‐cell RNA sequencing, FOXF1 expression was decreased in lung ECs in the mouse PARDS model. We used LEDD nanoparticles to restore FOXF1 by delivering a novel FOXF1‐activating compound, TanFe[F], which prevents FOXF1 protein degradation. LEDD‐TanFe[F] treatment increased FOXF1 protein and its activity in lung ECs in vitro and in vivo. LEDD‐TanFe[F] treatment was non‐toxic, well‐tolerated, and improved mice survival in the PARDS model. LEDD‐TanFe[F] reduced endothelial permeability, improved vascular repair, and alveolarization in injured neonatal lungs. LEDD‐supported drug delivery precisely to lung ECs is a promising therapeutic approach in PARDS.

## Graphical abstract

Pediatric acute respiratory distress syndrome (PARDS) suppresses the FOXF1 transcription factor and disrupts lung development. This study shows that the FOXF1‐activating small‐molecule compound, TanFe[F], can be precisely delivered to lung endothelial cells using the innovative LEDD nanoparticle system. The nano‐TanFe[F] therapy decreases lung inflammation, preserves endothelial function, and mitigates severe lung injury in a mouse model of PARDS.

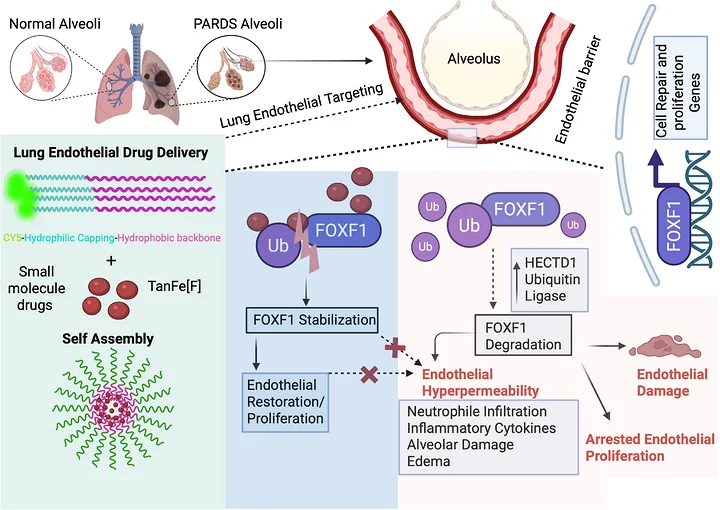

## 1 Introduction

Many new small molecule compounds for respiratory functions fail clinical trials due to unexpected toxicity. By the time therapeutic concentrations are achieved in the lung tissue, off‐target effects often become apparent, resulting in termination of clinical trials. Effective drug delivery to pulmonary endothelium in a lung‐specific manner remains a formidable challenge. Acute respiratory distress syndrome (ARDS) is a life‐threatening complication of acute lung injury (ALI) associated with inflammatory lung infiltrates, vascular and alveolar damage, leading to respiratory failure [1]. ARDS is a multifactorial disorder with major causes including pneumonia, burns, aspiration, transfusions, and sepsis; with pneumonia and sepsis being the most common causes [2], accounting for 30%–40% mortality rate in ARDS patients [3]. Hallmark of ARDS is endothelial dysfunction, which is associated with abnormal endothelial permeability leading to non‐cardiogenic pulmonary edema and increased ventilation‐to‐perfusion mismatch. Published studies reported that damage to the lung microvasculature results in pulmonary thrombosis [4] and subsequently alter the alveolar and vascular tree with decreased capillary density and lower alveolar counts observed both in human cases of ARDS and murine models [5]. Large observational study reported that 10.4% of intensive care unit (ICU) admissions were related to ARDS, and higher mortality was seen in cases that were under‐recognized and under‐treated at early stages [6].

With better understanding of the pathophysiological processes involved, it has become increasingly evident that the age of the populations with ARDS is important. Acute lung injury resulting in endothelial damage drives ARDS. However, the stressors can have differential effects in neonates, older kids and in adults in terms of epidemiology, outcomes, and response to available treatments. This may be the result of different cellular responses to the same stressors based on different maturation stages of the lungs and immune system. Recognizing these differences, specific clinical criteria have been utilized to diagnose ARDS in these populations and guide clinical management. The Pediatric Acute Lung Injury Consensus Conference Group has published the PALICC‐2 criteria to clinically define pediatric ARDS (PARDS) [7]. Despite the recognition of unique characteristics of the pediatric population, our understanding of the age‐specific‐molecular mechanisms involved is limited, and the management of such cases continues to be based on investigations predominately done in adult populations.

FOXF1 is a transcription factor essential for embryonic and postnatal lung development. FOXF1 is expressed in lung endothelial cells (ECs) where it orchestrates vascular repair after lung injury [8]. Recently, we used high‐throughput screening to identify the TanFe small molecule compound (Trans‐Activator of Nuclear FOXF1 Expression), which stabilizes the FOXF1 protein by protecting it from ubiquitination caused by HECTD1 E3 ubiquitin ligase [9]. Systemic TanFe treatment in utero increased FOXF1 protein levels and neonatal lung angiogenesis in newborn *Foxf1^+/−^* mice, a genetic model for Alveolar Capillary Dysplasia [9]. However, TanFe cannot be used for ARDS patients because the active site of TanFe has chlorine (Cl), which is highly reactive with oxygen, creating an important safety concern in ARDS patients requiring prolonged mechanical ventilation and supplemental oxygen. In the present study, we chemically modified the TanFe compound by replacing Cl with F (fluoride) and created a novel and non‐toxic TanFe derivative, TanFe[F], which we used for preclinical studies in a pediatric ARDS model.

Nanoparticle‐mediated delivery is a promising approach to transport therapeutic agents to the lung tissue. While intratracheal administration has been actively explored due to accumulation of therapeutic agents in lung tissue, cell‐specific drug delivery to injured pulmonary endothelium has never been achieved through the intratracheal route. A substantial body of work has explored endothelial‐specific targeting via the intravenous route using diverse carrier platforms such as liposomes, lipid nanoparticles, antibody conjugates, and polymeric nanocarriers. These carriers were often directed using EC markers, including PECAM‐1, ICAM‐1, and PV1/PLVAP [10, 11, 12]. These strategies have been validated in rodents, primates, and human cell culture models. However, reported endothelial‐targeting efficiencies were modest in these studies, and lung specificity has not been achieved with these carriers. Additionally, off‐target uptake by other cell types and organs was reported [13]. In the current study, we developed the Lung Endothelial Drug Delivery (LEDD) system, a specially designed formulation of Poly (β‐Amino Ester) (PBAE) nanoparticles with hydrophobic backbone capped with hydrophilic polyethylene glycol (PEG) tails, which is capable of universal drug delivery to the pulmonary endothelium through intravenous route. PBAE is a class of biodegradable polymers that offers excellent biocompatibility and low toxicity [14, 15, 16, 17], making it an ideal nanocarrier for drug delivery to the lung tissues. Therapeutic efficacy of the novel LEDD carrier with TanFe[F] cargo was investigated in a preclinical mouse model of PARDS.

## 2 Results

### 2.1 Single Cell RNAseq Analysis Reveals Decreased Expression of FOXF1 and Its Target Genes in Endothelial Cell Subsets in a Murine ARDS Model

To determine the effects of LPS injury on the transcriptome of pulmonary endothelial cells (ECs), we used single‐cell RNA sequencing of FACS‐sorted ECs obtained from lungs of mice injured by LPS, 2 days earlier (i.p. 12.5 mg/kg body weight). Using unbiased cell clustering analysis, we found five distinct EC populations in control and LPS‐injured groups. General capillaries (gCaps or CAP1), aerocytes (aCaps or CAP2), venous, arterial, and lymphatic ECs were identified using published markers (Figure 1A,B) [18, 19]. Our analysis focused on gCaps and aCaps because these microvascular ECs are leaky in this model [20, 21]. Gene ontology analysis revealed that LPS injury disrupted expression of genes linked to proangiogenic and anti‐apoptotic signaling (Figure 1C). Violin plots showed significant decreases in expression of the proangiogenic *Foxf1* gene and its transcriptional targets, including *Tmem100, Flt1, Tek, Aplnr, and Sema6*a, in LPS‐injured EC subsets (Figure 1D). In contrast, expression of *Hectd1* encoding the FOXF1‐inhibiting E3 ubiquitin ligase was increased after LPS injury (Figure 1D). Anti‐apoptotic gene *Bcl2* was selectively decreased in aCaps, whereas the anti‐apoptotic gene *Tmbim6* was decreased in both aCaps and gCaps (Figure 1D). Thus, LPS injury impairs normal EC gene signatures in the lung tissue.

**FIGURE 1:**
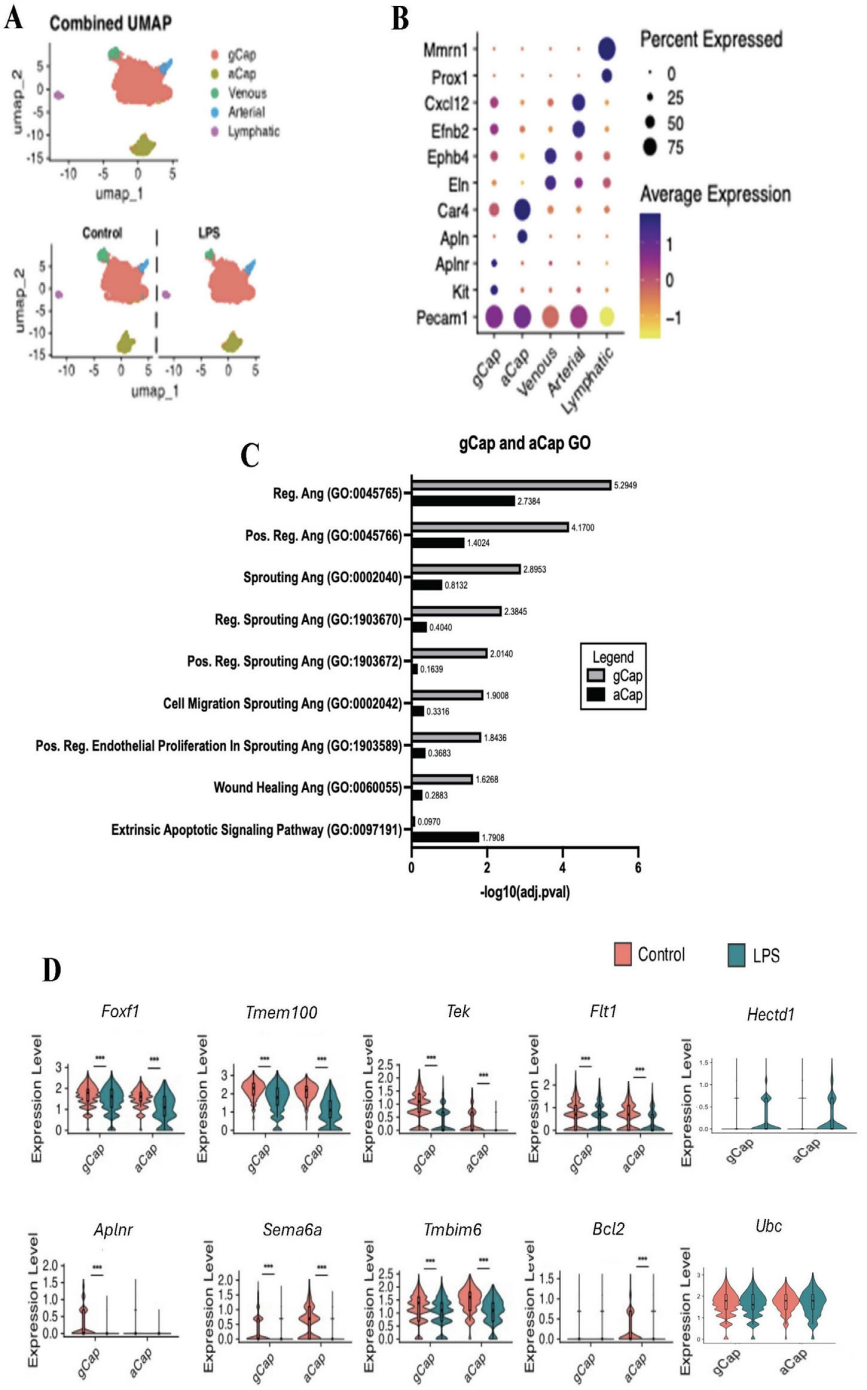
scRNAseq analysis of endothelial cells purified from lungs of mice treated with LPS. Datasets were generated from FACS‐sorted ECs obtained from lungs of mice treated with vehicle or LPS 2 days earlier. (A) Combined UMAP graph shows lung endothelial cell types from control and LPS‐injured mice (n = 13037 cells). Split UMAP graph shows ECs of control (n = 8273 cells) and LPS‐treated (n = 4764) mice. (B) Dotplot indicates EC genes used to identify EC clusters. Dot size corresponds to the number of cells expressing the corresponding gene. The color gradient of the dot from yellow to purple indicates expression levels, with yellow being the lowest and purple the highest. (C) Curated angiogenic and apoptotic gene ontology pathways identified in gCaps and aCaps. The X‐axis represents the ‐log10(adj.value), indicating the pathway significance. Numbers on the bars represent exact ‐log(adj.pval) values (‐log10(0.05) = 1.30). (D) Violin plots show expression of selected angiogenic and anti‐apoptotic genes from gCaps and aCaps. Orange represents control, while dark green indicates LPS treatment. Significance was calculated using a pairwise Wilcoxon test comparing control and LPS treated groups, with a Benjamin–Hochberg correction. The box within the plot represents the middle 50% of the data, with the lower edge showing the first quartile and the upper edge representing the third quartile. The line within the box shows the median expression of the data with whiskers extending from the minimum to the maximum of expression levels, *p* ≤ 0.0001 is ^***^.

### 2.2 TanFe[F] Increases the FOXF1 Protein in EC In Vitro

We recently used high‐throughput screening to identify the TanFe small‐molecule compound that increases the FOXF1 protein levels in ECs [9]. To improve stability and decrease toxicity of the compound, we chemically modified TanFe to produce a novel derivative, TanFe[F], which contains fluoride instead of Cl in the benzene ring (Figure 2A). Similar to TanFe [9], the newly synthesized TanFe[F] compound decreased protein‐protein interaction between FOXF1 and the HECTD1 E3 ubiquitin ligase, as shown by immunoprecipitation experiments (Figure S1). To determine the efficiency of TanFe[F] in increasing the FOXF1 levels, TanFe[F] was added to the cultured mouse lung ECs for 24 hrs. A dose‐dependent increase in the FOXF1 protein was observed in ECs treated with TanFe[F] as shown by Western blot (Figure 2B). Next, we examined the FOXF1 expression in ECs exposed to LPS treatment. LPS decreased the FOXF1 protein in ECs in vitro (Figure 2C), consistent with decreased FOXF1 expression in LPS‐injured lung ECs in vivo (Figure 1D). LPS‐mediated decline in FOXF1 levels was restored after treatment with TanFe[F] (Figure 2C). Next, we used the Nano‐Glo HiBiT Assay to test the effect of TanFe[F] on exogenous FOXF1 protein fused with the bioluminescent HiBiT tag. A human *FOXF1‐HiBiT* plasmid was generated and transfected into lung ECs. The transfected cells were treated with TanFe[F]. After 24 hrs, cells were harvested, and FOXF1‐HiBiT levels were analyzed using Luminescence‐based Nano‐Glo HiBiT Lytic Detection system. A significant increase in the FOXF1‐HiBiT protein was detected in transfected ECs treated with TanFe[F] compared to vehicle‐treated controls (Figure 2D). Luciferase activity was absent in ECs transfected with the empty plasmid (Figure 2E), demonstrating a high specificity of the Nano‐Glo HiBiT assay to the FOXF1‐HiBiT fusion protein. Altogether, TanFe[F] increases both endogenous and exogenous FOXF1 proteins in ECs in vitro.

**FIGURE 2:**
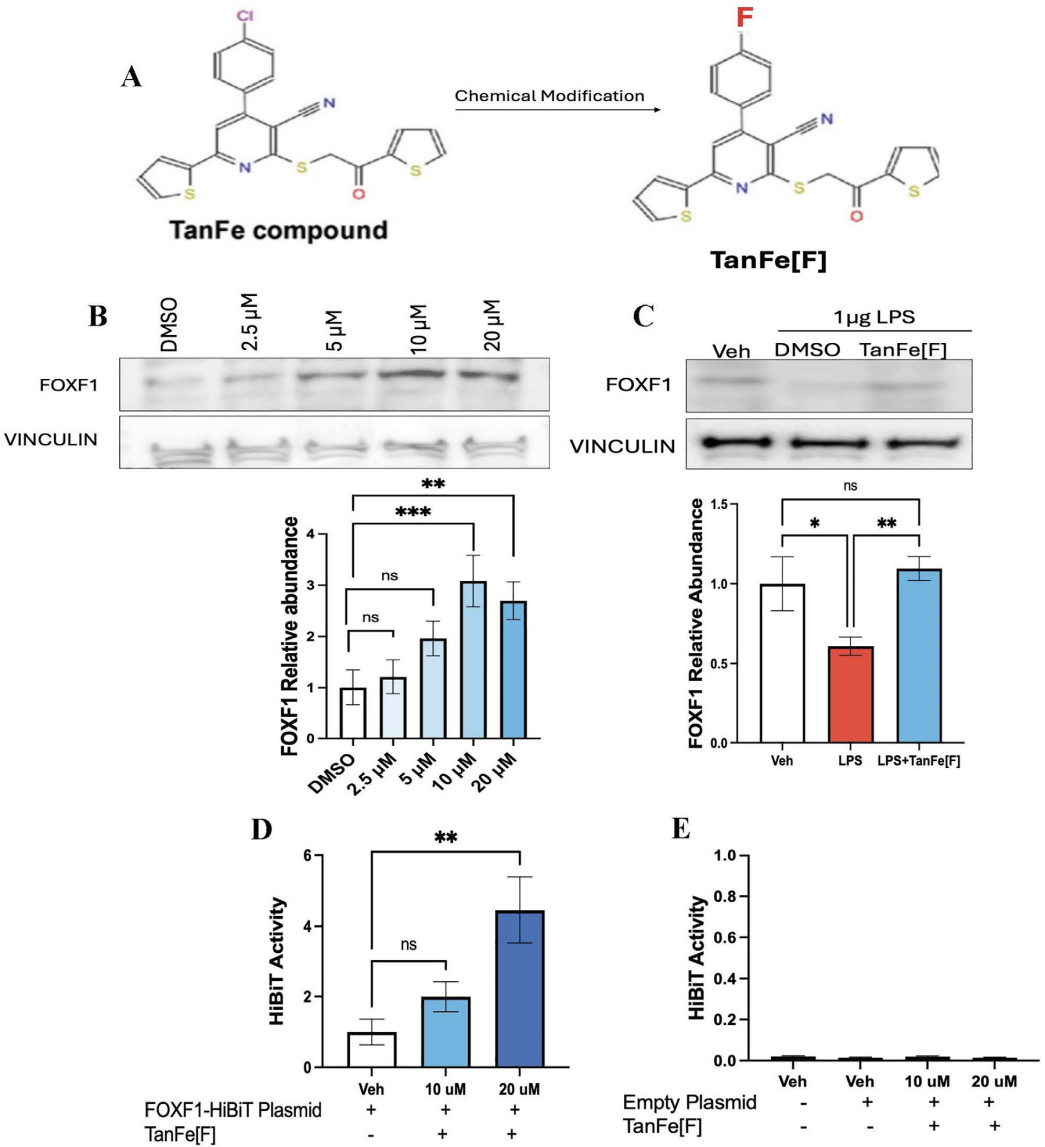
Dose‐dependent increase in FOXF1 protein abundance in MFLM‐91U cells treated with TanFe[F]. (A) Chemical structure of TanFe and TanFe[F] small molecule compounds. (B) MFLM‐91U endothelial cells were treated with vehicle or varying concentrations of TanFe[F] for 24 hrs. Western blot analysis indicates an increase in FOXF1 protein amounts in cells treated with higher concentrations of TanFe[F]. Protein expression of FOXF1 was normalized to Vinculin to quantify the relative FOXF1 abundance (n = 3/group). (C) FOXF1 protein is decreased in LPS‐treated cells and reversed back to normal after TanFe[F] exposure. Protein abundance of FOXF1 was normalized to Vinculin (n = 3/group). (D, E) MFLM‐91U cells were transfected with the plasmid containing HiBiT‐tagged human *FOXF1* cDNA or empty plasmid. Twenty‐four hours following the transfection, cells were treated with either vehicle or TanFe[F] (10 µM or 20 µM). Bioluminescence was measured 24 hrs after the drug exposure. Statistical significance was calculated using one‐way ANOVA with Tukey's post hoc multiple comparisons. Data show mean ± SD, n = 3 cell cultures per group, *p* ≤ 0.05 is ^*^, *p* ≤0.01 is ^**^, ns is not significant.

### 2.3 Developing Nanoparticle Formulation Capable of Delivering TanFe[F] to Lung ECs In Vivo

The TanFe[F] compound is highly hydrophobic, making its delivery in vivo challenging. We recently developed nanoparticles made from PBEA, PEI, and PEG polymers that can deliver DNA plasmids into pulmonary ECs, but not to other cells or organs [14]. Despite this specificity, these nanoparticles cannot deliver the TanFe[F] compound due to its physiochemical properties. To address this problem, we designed and synthesized three new formulations of polyplex nanoparticles (NPs) capable of holding TanFe[F] inside the hydrophobic core (Figure 3A). Among these nanoparticles are short PBAE backbone NPs (PBAE‐1.6DA‐PEG1X), long backbone NPs (PBAE‐1.4DA‐PEG1X), and PBAE backbone with higher PEG content (PBAE‐1.4DA‐PEG4X).

**FIGURE 3:**
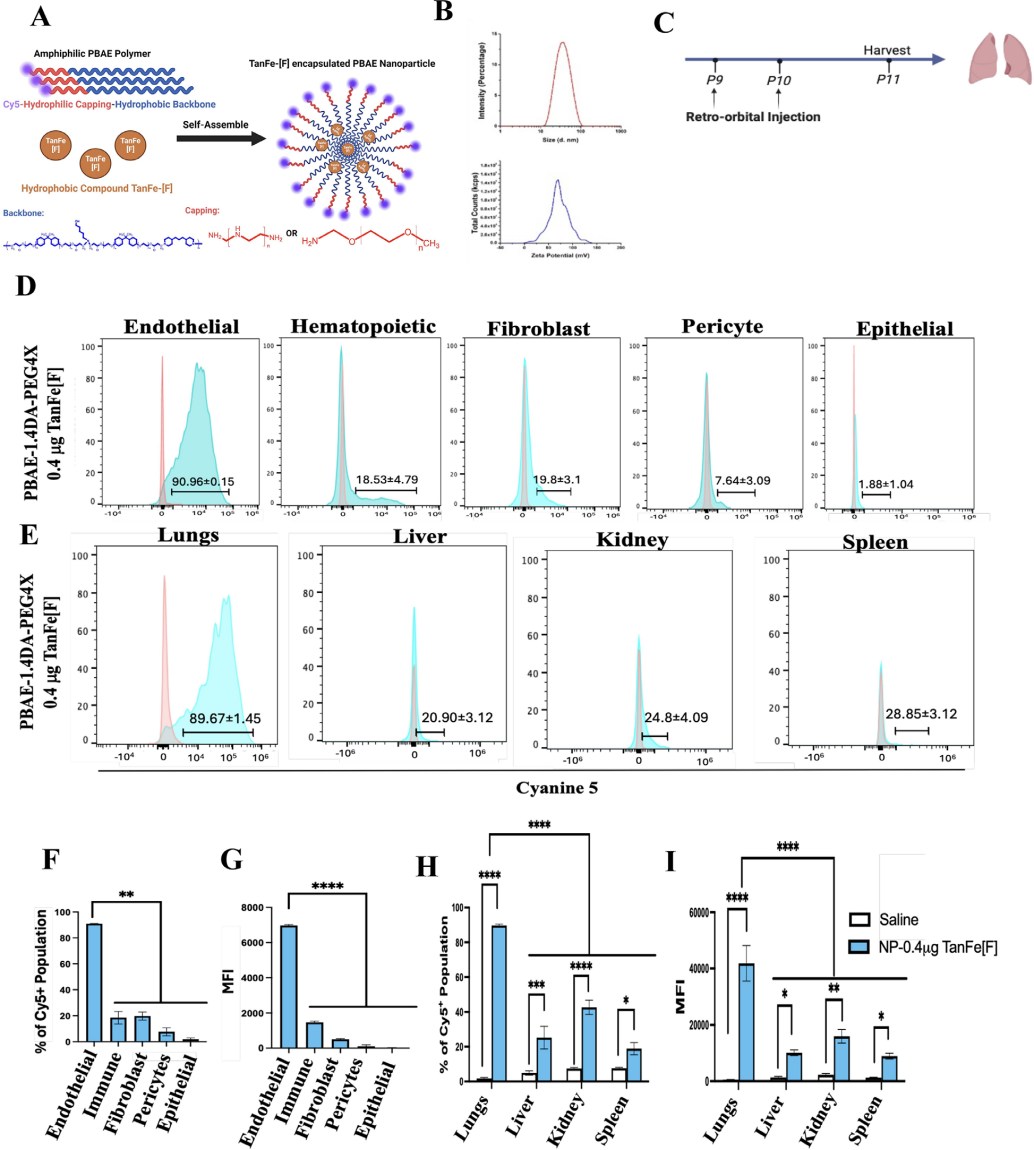
PBAE‐1.4DA‐PEG4X nanoparticles specifically deliver TanFe[F] to lung ECs but not to the ECs of other organs. (A) 0.4 µg of TanFe[F] compound was encapsulated into PBAE‐1.4DA‐PEG4X nanoparticles labeled with Cyanine 5 (Cy5). (B) The size and charge of PBAE‐1.4DA‐PEG4X nanoparticles containing TanFe[F] were determined using Malvern Zetasizer Ultra. (C) Nano‐TanFe[F] treatment was performed in C57BL6 mice using retro‐orbital injection. (D) Flow cytometry analysis of different cell types in the lung tissue or (E) ECs from different organs shows presence of nanoparticles with TanFe[F] (blue curve). Red curve shows flow cytometry of ECs from untreated mice. (F) Percentages of Cy5^+^ cells and (G) Mean fluorescent intensity (MFI) show EC‐selective targeting after TanFe[F] treatment. (H) Percentages of Cy5^+^ ECs and (I) MFI in ECs from different organs show lung‐specific EC targeting. Statistical significance was calculated using one‐way or two‐way ANOVA with Tukey's post hoc multiple comparisons. Data show mean ± SD, n = 3 mice per group, *p* ≤ 0.05 is ^*^, *p* ≤ 0.01 is ^**^, *p* ≤ 0.001 is ^***^, *p* ≤ 0.0001is ^****^, ns is not significant.

The hydrodynamic diameter of the PBAE‐1.4DA‐PEG4X nanoparticles containing TanFe[F] was 33.66 ± 0.336 nm, and the polydispersity index (PDI) was 0.1389 ± 0.018 (Figure 3B). The surface charge of the nanoparticles was 65.21 ± 5.619 mV (Figure 3B), providing a positive NP charge ideal for binding to the negatively charged EC glycocalyx. Cyanine 5 (Cy5)‐labeled NP‐TanFe[F] complexes were intravenously (i.v.) injected to mice at postnatal (P) day 9 and P10 through the retro‐orbital sinus (Figure 3C). At P11, lungs and other organs were harvested to identify cell types containing the nanoparticles by flow cytometry (Figure 3D,E). In the lungs, nanoparticles with higher PEG content (PBAE‐1.4DA‐PEG4X) mainly targeted ECs (CD31^+^CD45^−^), with minimal targeting of other cell types such as hematopoietic cells (CD45^+^CD31^−^), fibroblasts (CD140a^+^CD31^−^CD45^−^), epithelial cells (CD326^+^CD31^−^CD45^−^CD140a^−^) and pericytes (NG2^+^CD326^−^CD31^−^CD45^−^CD140a^−^) (Figure 3D,F). Mean fluorescent intensity (MFI) was also higher in lung ECs compared to other cell types (Figure 3G), indicating a higher NP load per cell. The targeting efficiency and specificity of NP formulations with low PEG contents PBAE‐1.4DA‐PEG1X and PBAE‐1.6DA‐PEG1X was lower compared to the PBAE‐1.4DA‐PEG4X formulation (Figures S2–S4A,B), despite they had comparable hydrodynamic diameter and zeta potential (Figure S4C). Therefore, we have chosen the PBAE‐1.4DA‐PEG4X for the subsequent studies. We have also characterized PBAE‐1.4DA‐PEG4X nanoparticles for encapsulation efficiency, release profile, serum stability, and cellular uptake. Data indicate efficient encapsulation and release as shown by standard curve and time course studies (Figure S5A,B). The nanoparticles were stable in serum with the hydrodynamic size remaining small and no significant aggregation (Figure S5C, left panel). In the presence of serum, the size distribution showed a peak under 10 nm corresponding to serum proteins, while the nanoparticles maintained uniform distribution (Figure S5C, right panel). Zeta potential in saline was approximately 20 mV (Figure S5D), providing a suitable surface charge for targeting the endothelium (Figure S5D). Furthermore, efficient uptake of nanoparticles was observed in endothelial cells in vitro (Figure S5E).

Next, we directly compared the presence of nanoparticles with TanFe[F] in ECs of the lung, liver, spleen, and kidney. The NP‐TanFe[F] complexes exhibited lung endothelial‐selective targeting after intravenous administration as shown by flow cytometry (Figure 3E,H,I). NPs with either 0.4 or 1 µg of TanFe[F] per injection exhibited similar lung EC selectivity (Figure 3D–I and Figure S6A–D). We also developed a negative control nanoparticle formulation using simple PBAE polymers with linear backbones and a single capping agent. The negative control NPs encapsulated with 0.4 µg of TanFe[F] were directly compared with TanFe[F] containing PBAE‐1.4DA‐PEG4X NPs after i.v. delivery (Figure S7A). The PBAE‐1.4DA PEG4X NPs exhibited a robust endothelial targeting compared to the control (Figure S7A,B).

Importantly, the PBAE‐1.4DA‐PEG4X nanoparticles can encapsulate and specifically deliver to lung endothelium other hydrophobic small‐molecule compounds, such as Vorinostat, RCM‐1, and TanFe (Figure S8A–D). Therefore, the PBAE‐1.4DA‐PEG4X NPs can be used as Lung Endothelial Drug Delivery system (LEDD) for therapeutic applications.

### 2.4 Nanoparticle‐TanFe[F] Treatment Increases the FOXF1 Protein Level In Vivo

To determine if the nano‐TanFe[F] treatment increases the FOXF1 protein in vivo, mice were injected with human *FOXF1‐HiBiT* plasmid encapsulated into PBAE‐P22‐F1 nanoparticles for DNA delivery [14]. Twenty‐four hours after *FOXF1‐HiBiT* plasmid delivery, mice were given LEDD nanoparticles containing the TanFe[F] small molecule compound. Mice were harvested 24 hrs after the nano‐TanFe[F] treatment (Figure 4A), and the single‐cell suspension was incubated with Nano‐Glo HiBiT lytic reagent containing the substrate and LgBiT protein, detecting the exogenous FOXF1‐HiBiT protein transcribed from the plasmid (Figure 4B,C). Bioluminescence was higher in the lungs of mice treated with *FOXF1‐HiBiT* and nano‐TanFe[F] compared to mice treated with *FOXF1‐HiBiT* alone (Figure 4D). Thus, TanFe[F] increases the FOXF1‐HiBiT protein in lung tissue. We also used transgenic mice expressing green fluorescence protein (GFP) under the control of FOXF1 promotor [18] to examine FOXF1 expression in pulmonary endothelial cells. Nano‐TanFe[F] treatment increased the percentage of FOXF1‐expressing (GFP^+^) endothelial cells compared to saline‐treated mice (Figure 4E,F and Figure S9).

**FIGURE 4:**
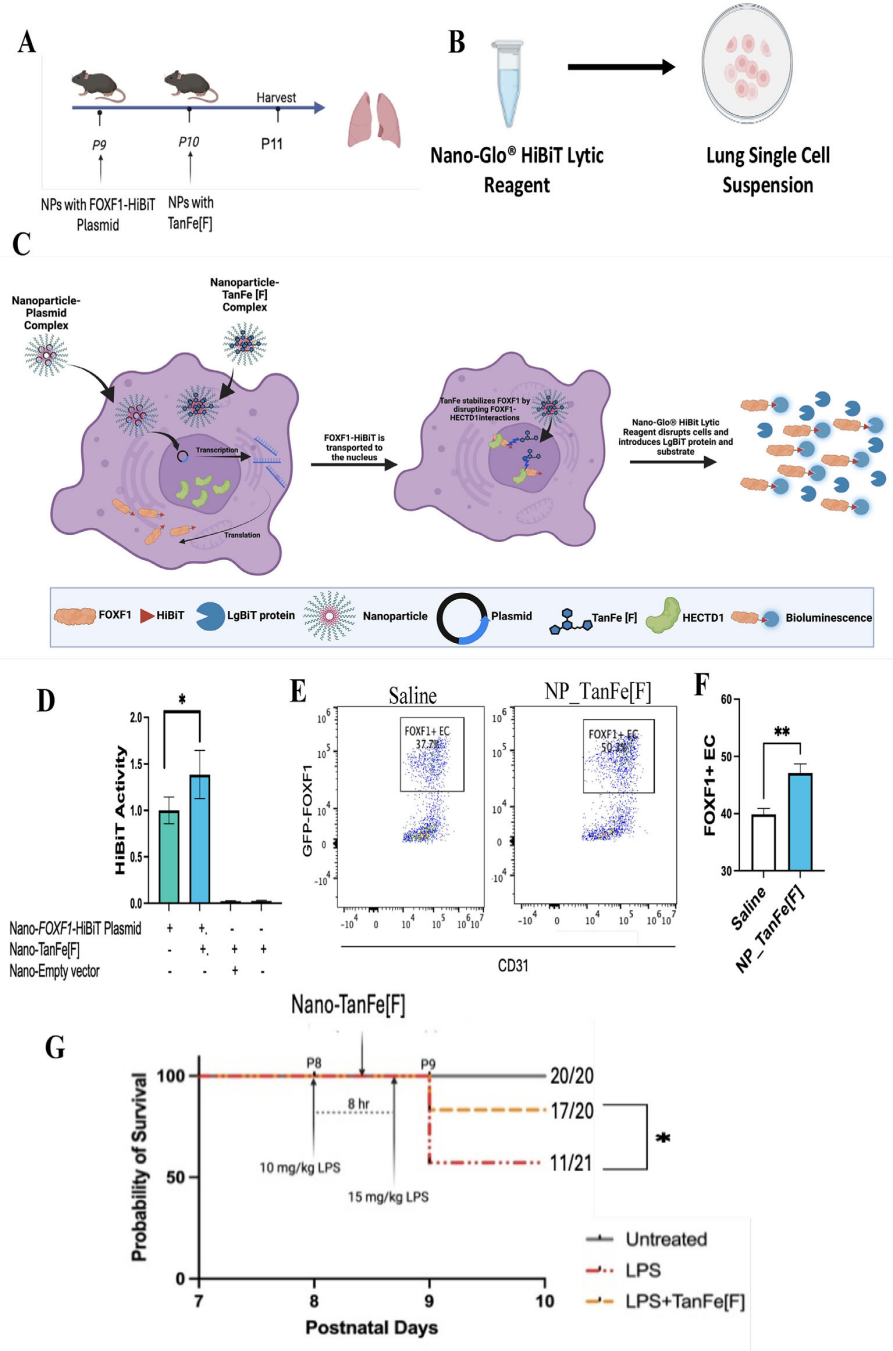
Nanoparticle‐delivery of TanFe[F] increases FOXF1 protein in the lung tissue and improves survival of LPS‐injured mice. (A) C57BL6 mice were treated with nanoparticles containing a plasmid with HiBiT‐tagged human *FOXF1* cDNA on P9. Nanoparticles with TanFe[F] was delivered on P10. (B) Mouse lungs were harvested on P11 to prepare single‐cell suspensions, which were incubated with HiBiT lytic reagent. (C) Schematic that shows FOXF1‐HiBiT fusion protein increases when in contact with large HiBiT (LgBiT) and substrate to produce bioluminescence. (D) HiBiT activity was higher in lungs of mice treated with FOXF1‐HiBiT and nano‐TanFe[F] compared to lungs treated with FOXF1‐HiBiT alone. Statistical significance was calculated using Welch t‐test. Data show mean ± SD, n = 4–6 mice per group, *p* ≤ 0.05 is ^*^. (E, F) Transgenic mice expressing green fluorescence protein (GFP) under the control of FOXF1 promotor were treated with either saline or nanoparticle with TanFe[F] on P9‐10. Mice were harvested on P11 for flow cytometry to quantify FOXF1^+^ endothelial cells (CD45^−^CD31^+^ GFP^+^). Percentage of FOXF1^+^ endothelial cells was shown as mean ± SD (n = 4 mice per group). Statistical significance was calculated using a Welch t‐test. *p* ≤ 0.01 is ^**^. (G) Kaplan–Meier curves show a lower survival rate in mice treated with 2 doses of LPS compared to untreated mice. Survival rate was improved in LPS‐treated mice after TanFe[F] treatment, n = 20–21 mice per group, ^*^ is logrank *p*‐value ≤ 0.05.

### 2.5 Nanoparticle‐TanFe[F] Treatment Improves Mice Survival After LPS Injury

To determine the survival rate in mice receiving the nano‐TanFe[F] treatment, mice were challenged with two intraperitoneal (i.p.) doses of LPS given with 8‐hr interval. These two doses of LPS were used to achieve the LD50 needed for survival studies in a mouse model of PARDS.

Four hours after the first LPS dose, mice were injected i.v. with nano‐TanFe[F] using the LEDD delivery system. The nano‐TanFe[F] treatment increased the survival rate in LPS‐injured mice from 52% to 85% (Figure 4G).

To evaluate the safety of the nano‐TanFe[F] complexes, uninjured mice were i.v. injected twice with nanoparticles containing TanFe[F]. Mice were harvested 24 hrs after the second injection to collect the blood for toxicological and hematological evaluation. Basic metabolic parameters were within the normal range (Figure S10A). The total protein, albumin, globulin, and total bilirubin were similar in the blood serum of untreated and nano‐TanFe[F] treated mice (Figure S10A,B), as were differential cell counts in the peripheral blood (Figure S10C,D). Histological analysis of major organs (kidney, liver, and spleen) showed no observable tissue damage, inflammation or structural changes after nanoparticle treatment (Figure S10E). Thus, two nano‐TanFe[F] treatments were not associated with adverse biochemical, hematologic, and morphological outcomes.

### 2.6 Nanoparticle Delivery of TanFe[F] Restores the Weight Loss After LPS Injury

Since two nano‐TanFe[F] treatments were nontoxic and well tolerated, we tested their therapeutic efficacy in a pediatric mouse ARDS model. Since two i.p. doses of 10 mg/kg of LPS resulted in a low survival rate (Figure S11), we decreased the dose to 6 mg/kg of LPS administered at P7 and P8. LPS‐injured pups were treated with nano‐TanFe[F] at P9 and P10 through the i.v. route (Figure 5A). Throughout the exposure, weights of mice were recorded. While uninjured mice continued to gain weight, there was a significant decline in the body weight of LPS‐injured mice (Figure 5B). Nano‐TanFe[F] treatment improved the weight gain of LPS‐injured mice as early as P10 (Figure 5B). Treatment with empty nanoparticles had no effect on body weights in uninjured or LPS‐injured mice (Figure 5B). In human pulmonary microvascular endothelial cells (HPMEC) in vitro, LPS treatment induced the expression of proinflammatory (*IL1β*) mRNA. But decreased *S1PR1* and *APLNR* mRNAs. TanFe[F] treatment restored expression levels of these genes in LPS‐treated human lung endothelial cells (Figure S12).

**FIGURE 5:**
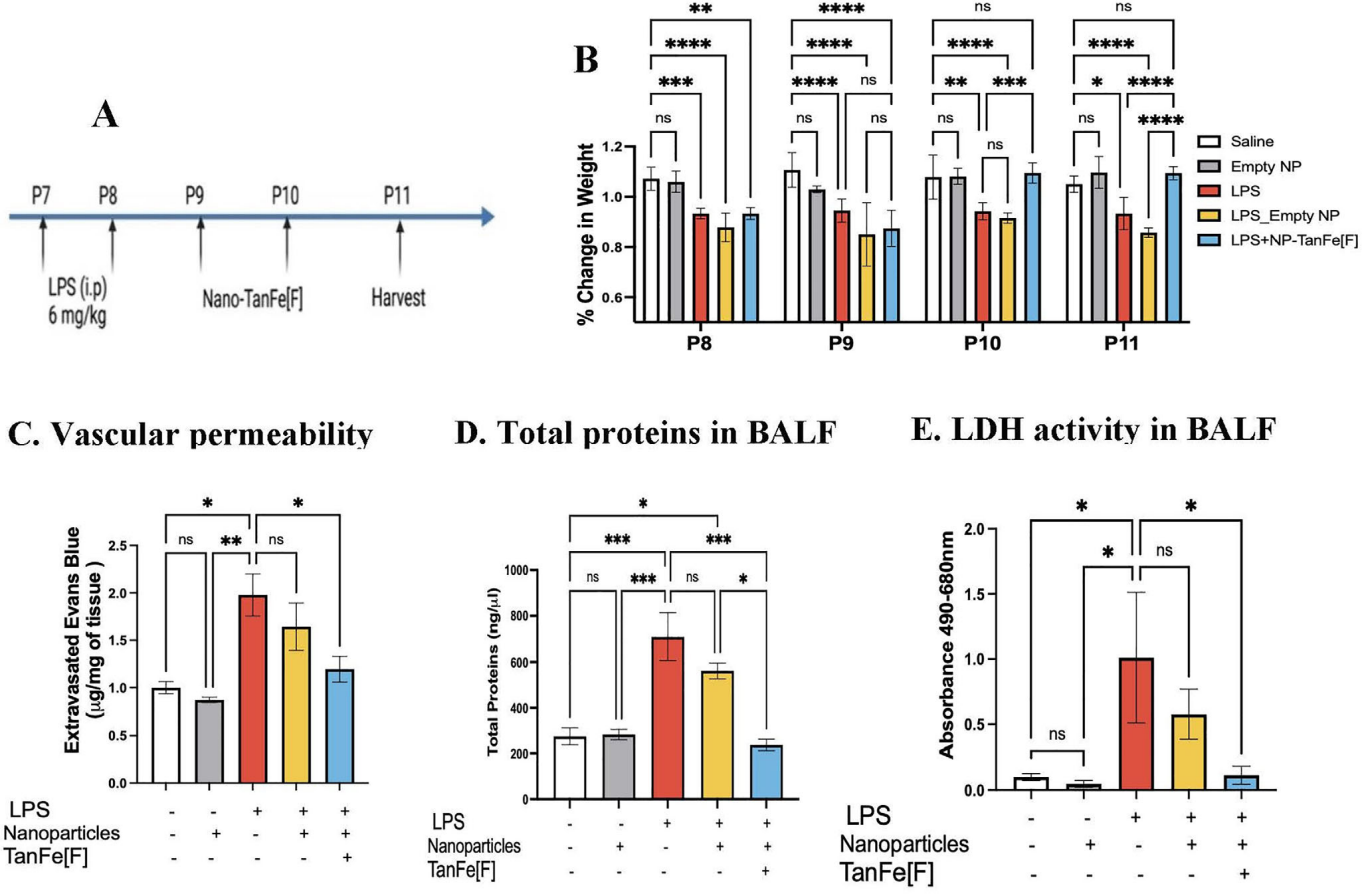
Nano‐TanFe[F] treatments protect LPS‐induced weight loss and endothelial barrier disruption. (A) C57BL6 mice were treated with 6 mg/kg LPS on P7 and P8. Following LPS treatment, mice were randomized and treated with either empty nanoparticles or nanoparticles with 0.4 µg of TanFe[F] on P9 and P10. (B) Reduced body weight is seen in mice receiving LPS 24 hrs after the injury. Weight gain was restored in LPS‐injured mice receiving 0.4 µg of TanFe[F] 24 hrs after nano‐TanFe[F] treatment. Statistical significance was calculated using two‐way ANOVA with Tukey's post hoc multiple comparisons. Data are shown as mean percent weight change ± SD, n = 8–10 mice per group. (C) Nano‐TanFe[F] treatments protect the EC barrier function in LPS‐treated mice. Evans blue dye was injected retro‐orbitally. Four hours after the injection, lungs were thoroughly perfused and harvested to quantify the extravasated Evans blue dye. (D, E) Total protein and LDH activity were measured in BALF of uninjured, LPS‐injured, and nano‐TanFe[F]‐treated mice. Statistical significance was calculated using one‐way ANOVA with Tukey's post hoc multiple comparisons. Data show mean ± SD, n = 3–5 mice per group, *p* ≤ 0.05 is ^*^, *p* ≤ 0.01 is ^**^, *p* ≤ 0.001 is ^***^, *p* ≤ 0.0001 is ^****^, ns is not significant.

### 2.7 Nanoparticle Delivery of TanFe[F] Restores Lung Endothelial Barrier Function and Attenuates Lung Inflammation After LPS Injury

Increased endothelial permeability in lung tissue is a key hallmark of ARDS [9, 22]. Endothelial permeability was increased in LPS‐injured lungs as shown by extravasated Evans blue dye (Figure 5C). Nano‐TanFe[F] treatment reduced endothelial permeability after LPS injury, whereas empty nanoparticles had no effect (Figure 5C). Total protein and LDH activity, both biomarkers of lung injury [23], were increased in broncho‐alveolar lavage fluid (BALF) of LPS‐injured mice (Figure 5D,E). Nano‐TanFe[F] treatment decreased total BALF protein and LDH activity after LPS injury, whereas empty nanoparticles had no effect in uninjured or LPS‐injured mice (Figure 5D,E).

To examine pulmonary inflammation, a hallmark of pediatric ARDS [24], we evaluated differential cell counts in BALF using cytospin (Figure 6A). Nano‐TanFe[F] treatment decreased the numbers of macrophages and neutrophils in BALF (Figure 6B,C) and reduced the expression of *Tnfα* and *Il1β* in the lung tissue of LPS‐injured mice (Figure 6D). Treatments with empty nanoparticles had no effect (Figure 6B–D). Altogether, nano‐TanFe[F] treatment via the LEDD system restores endothelial barrier function and attenuates lung inflammation after LPS injury.

**FIGURE 6:**
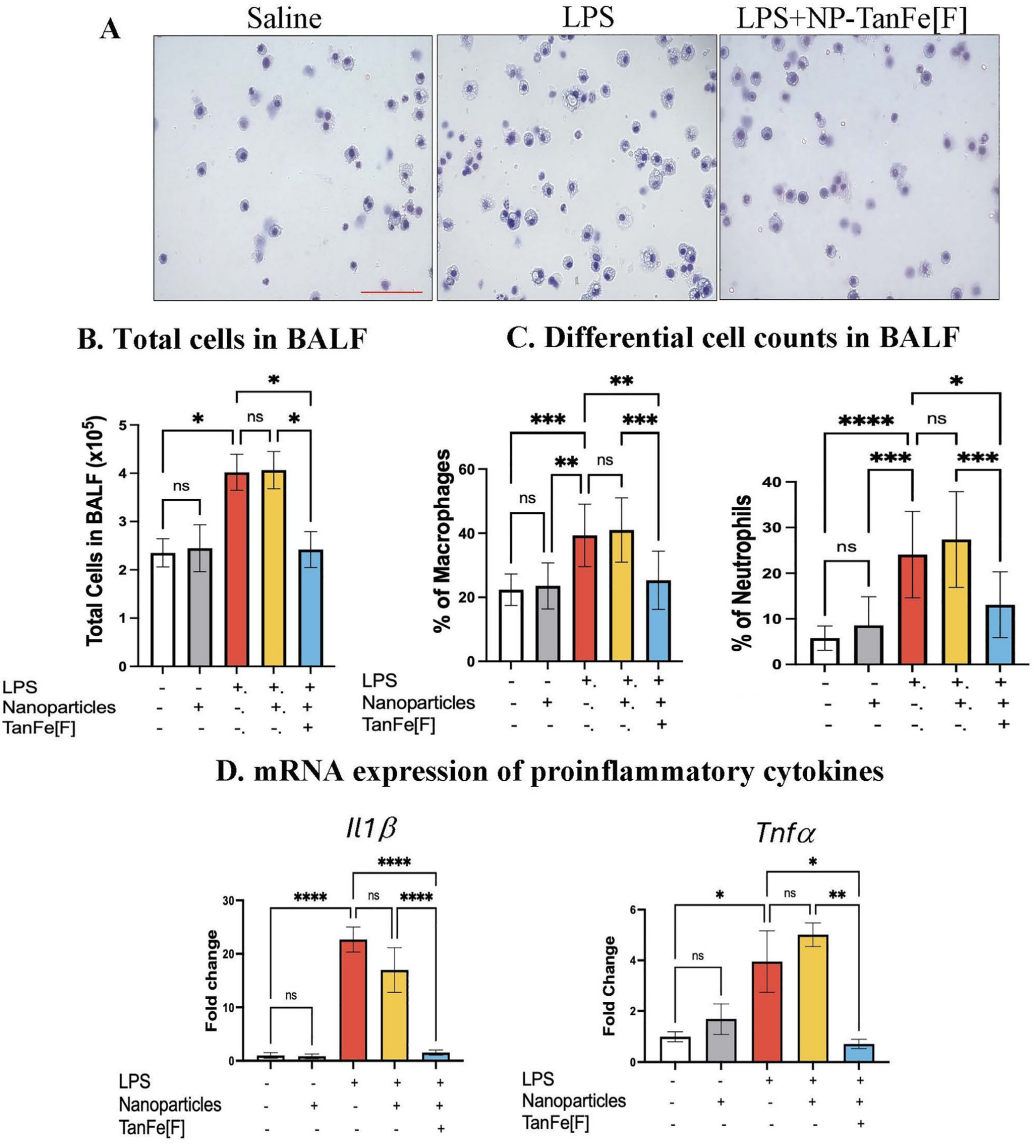
Nano‐TanFe[F] treatments protect LPS‐treated neonatal mice from pulmonary inflammation. (A) BALF was collected and stained for differential cell counting. Bright‐field images show increased inflammation after LPS injury and the anti‐inflammatory effect of nano‐TanFe[F] treatments. The scale bar is 50 microns. (B, C) Total cell counts and percentages of macrophages and neutrophils in BALF were quantified and show anti‐inflammatory benefits of nano‐TanFe[F] treatments (n = 5–7 mice per group). (D) mRNA expression of *Il1β* and *Tnfα* in whole lung tissue were quantified using quantitative real‐time RT‐PCR (n = 4–6 mice per group). Statistical significance was calculated using one‐way ANOVA with Tukey's post hoc multiple comparisons. Data show mean ± SD, *p* ≤ 0.05 is ^*^, *p* ≤ 0.01 is^**^, *p* ≤ 0.0001 is ^****^, ns is not significant.

### 2.8 Nanoparticle Delivery of TanFe[F] Increases Numbers of ECs and Endothelial Progenitor Cells After LPS Injury

LPS‐injured mice were treated with 2 doses of nano‐TanFe[F] after the injury, and lungs were harvested on P14 (Figure 7A). Immunostaining for FOXF1 and CD31 was used to evaluate the FOXF1^+^ endothelial population in treatment groups. FOXF1 was abundantly expressed in ECs of uninjured mouse lungs (Figure 7B,C and Figure S13A), consistent with published studies [25]. LPS injury reduced the percentage of FOXF1^+^ ECs, whereas the nano‐TanFe[F] treatment prevented the decline of ECs in LPS‐injured lungs (Figure 7B,C). Since FOXF1 stimulates endothelial proliferation [25, 26], co‐staining for Ki‐67 and CD31 was used to examine the number of proliferating ECs. The percentage of Ki‐67^+^ ECs was lower in LPS‐injured neonatal lungs (Figure 7D and Figure S13B,C), consistent with the disruption of normal lung developmental processes. Nano‐TanFe[F] treatment restored the percentage of Ki‐67^+^ ECs after LPS injury (Figure 7D). Based on qRT‐PCR of total lung RNA, LPS injury decreased mRNAs of EC genes such as *Erg* and *Tmem100* (Figure 7E). Nano‐TanFe[F] treatment restored the expression of these EC genes in LPS‐injured lungs. (Figure 7E). Treatments with empty nanoparticles did not change EC proliferation or expression of EC genes (Figure 7E).

**FIGURE 7:**
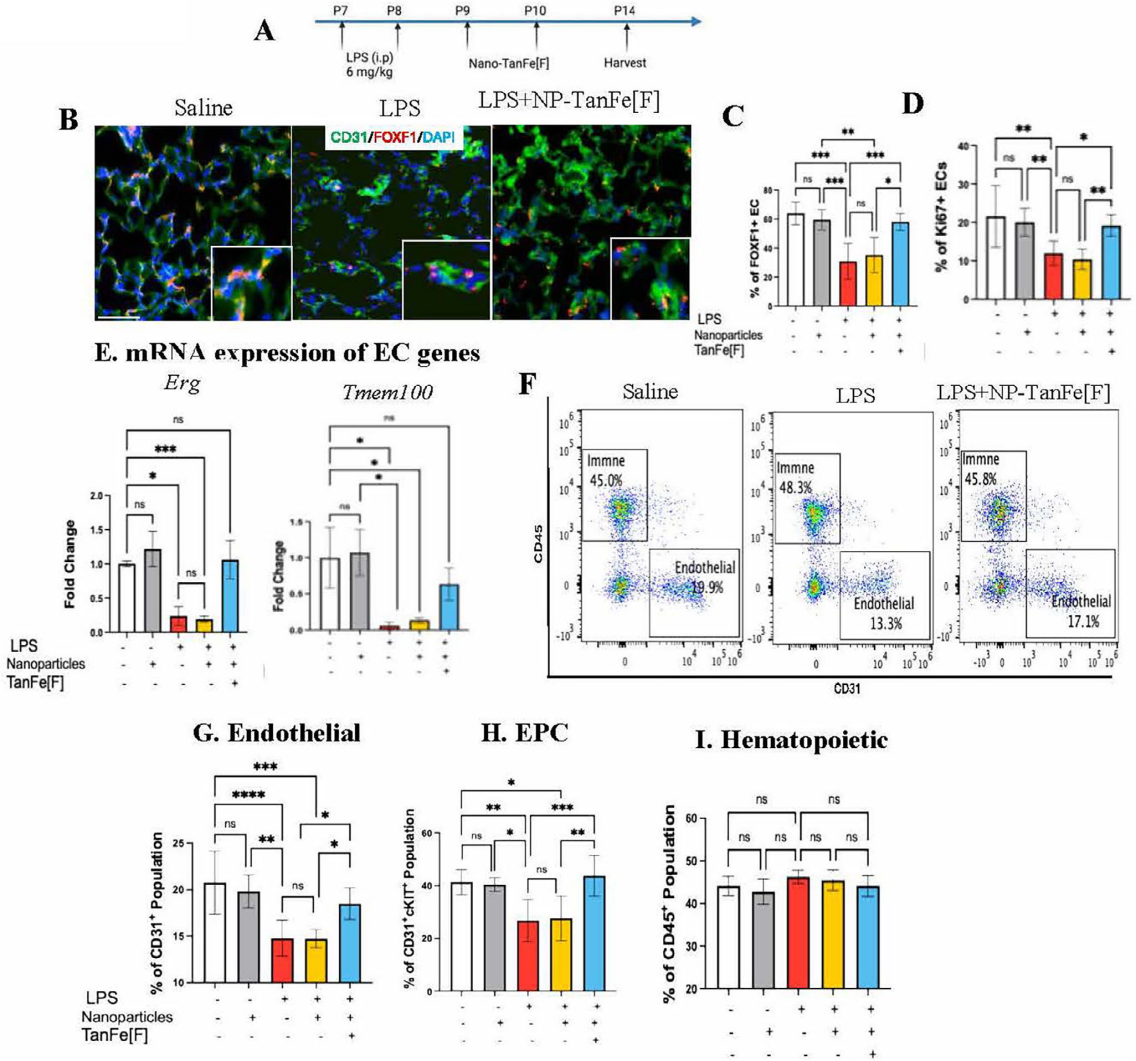
Nano‐TanFe[F] treatments increase EC proliferation in LPS‐injured neonatal mice lungs. (A) C57BL6 mice were injured with 6 mg/kg LPS at P7 and P8. Mice were randomized and treated with either empty nanoparticles or nanoparticles containing 0.4 µg of TanFe[F]. Lungs were harvested at P14. (B) Frozen lung sections were stained with antibodies against CD31 and FOXF1. DAPI was used to stain cell nuclei (blue). The scale bar is 50 microns. (C) The percentage of FOXF1^+^ ECs was quantified using 8 random images from 3 mice in each group. (D) The percentage of Ki‐67^+^ ECs was quantified using 8 random images from 3 mice in each group (E) mRNAs of EC‐specific genes *Erg* and *Tmem100* were quantified in whole lung tissue using quantitative real‐time RT‐PCR (n = 6–8 mice per mice per group). (F,I) Lungs were analyzed using flow cytometry to quantify hematopoietic cells (CD31^−^CD45^+^), endothelial cells (ECs; CD45^−^CD31^+^) and endothelial progenitor cells (EPCs; CD45^−^CD31^+^cKIT^+^). Percentages of ECs, EPCs, and hematopoietic cells in the lung tissue were shown as mean ± SD (n = 5–7 mice per group). Statistical significance was calculated using one‐way ANOVA with Tukey's post hoc multiple comparisons. Data are shown as mean ± SD, *p* ≤ 0.05 is^*^, p≤ 0.01 is^**^, *p* ≤ 0.001 is ^***^, *p* ≤ 0.0001 is ^****^, ns is not significant.

Next, we used flow cytometry to quantitate the total number of ECs, endothelial progenitor cells (EPCs), and hematopoietic cells in lung tissues. Single‐cell suspension was prepared from enzymatically digested lungs and stained with anti‐CD45, anti‐CD31, and anti‐cKIT antibodies to identify hematopoietic cells (CD31^−^CD45^+^), endothelial cells (CD31^+^CD45^−^), and endothelial progenitor cells (cKIT^+^ CD31^+^ CD45^−^), consistent with published studies [25]. LPS injury decreased the number of ECs and EPCs in the lung tissue (Figure 7F and Figure S14A,B). LPS‐induced declines in both cell populations were prevented after nano‐TanFe[F] treatment (Figure 7G,H). Interestingly, there was no significant difference in the number of CD45^+^ cells in the lung tissue at P14 (Figure 7I), consistent with published studies demonstrating that leukocyte recruitment quickly declines in neonatal lungs after LPS injury [27]. Thus, nano‐TanFe[F] treatment prevents the loss of lung ECs and EPCs in a mouse model of pediatric ARDS.

### 2.9 Nanoparticle‐Delivery of TanFe[F] Improves Alveolarization and Increases the Capillary Density in LPS‐Injured Mouse Lungs

Since pediatric ARDS leads to decreased alveolarization and capillary density in alveolar regions [3], we evaluated the lung structure in LPS‐injured mice treated with nano‐TanFe[F] (Figure 8A). At P14, LPS‐injured mice exhibited alveolar simplification (Figure 8B). Nano‐TanFe[F] treatment prevented the alveolar simplification as shown by a reduction in the percentage of airspaces (Figure 8A,B). Furthermore, LPS injury decreased the capillary density as shown by endomucin immunostaining (Figure 8C,D), consistent with decreased numbers of ECs and EPCs found by flow cytometry (Figure 7G,H). Nano‐TanFe[F] treatment increased the alveolar capillary density after LPS injury (Figure 8C,D). Altogether, nanoparticle TanFe[F] treatment via the LEDD system improves neonatal angiogenesis and alveolarization in a mouse model of pediatric ARDS.

**FIGURE 8:**
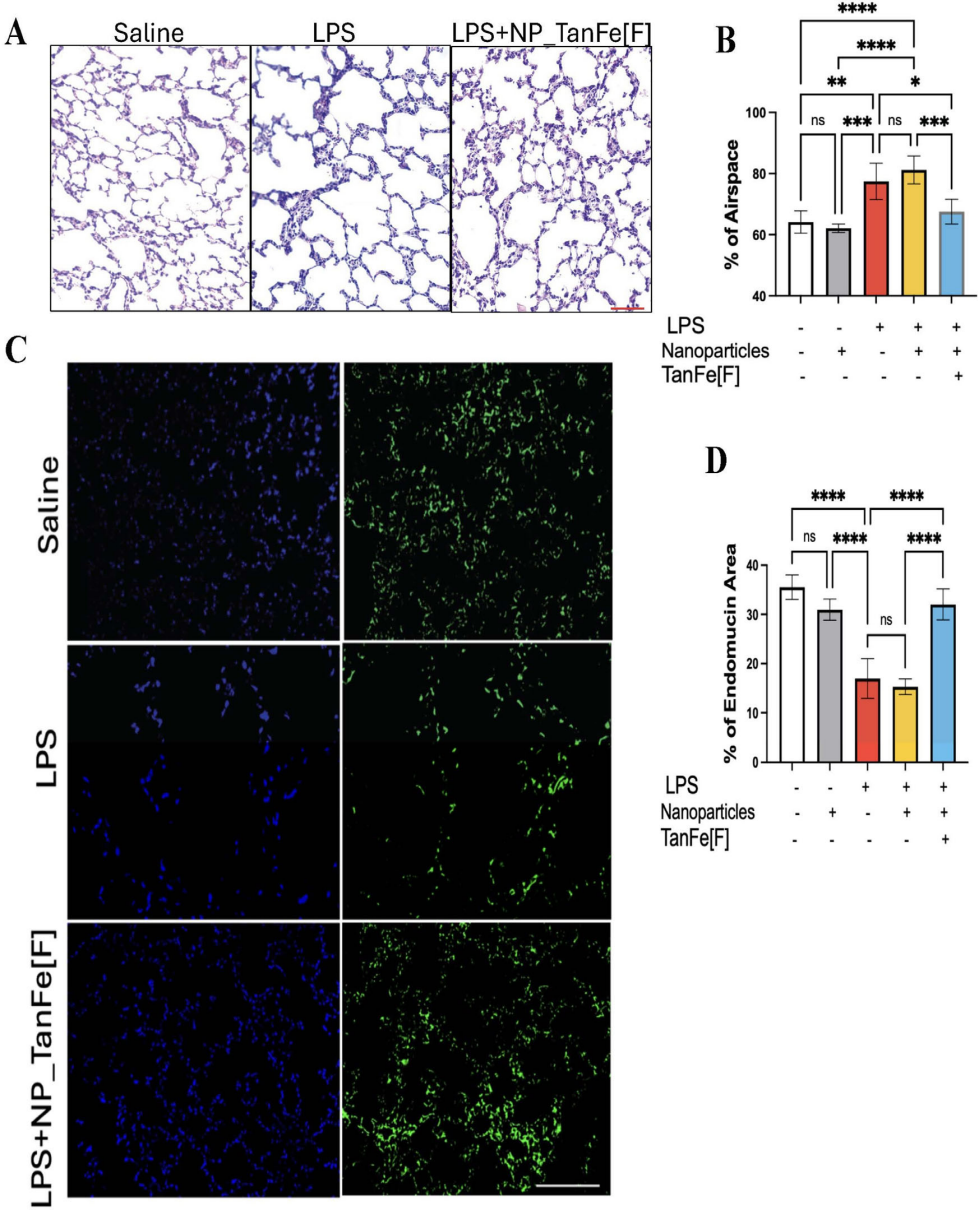
Nano‐TanFe[F] treatments improve alveolarization and increase the capillary density in LPS‐injured neonatal mouse lungs. C57BL6 mice were treated with 6 mg/kg LPS at P7 and P8. Mice were randomized and treated with either empty nanoparticles or nanoparticles encapsulated with 0.4 µg of TanFe[F]. Lungs were harvested at P14. (A) Lung sections were stained with H&E. The scale bar is 50 microns. (B) The airspace quantification was performed by ImageJ using 8 random images from 3 mice in each group. (C) Immunostaining for Endomucin was performed to determine the capillary density in alveolar regions. DAPI was used to stain cell nuclei (blue). The scale bar is 50 microns. (D) The percentage of endomucin‐positive area was quantified by ImageJ using 8 random images obtained from 3 mice in each group. Statistical significance was calculated using one‐way ANOVA with Tukey's post hoc multiple comarisons. Data are shown as mean ± SD, n = 3 mice per group, *p* ≤ 0.05 is ^*^, *p* ≤ 0.01 is ^**^, *p* ≤ 0.001 is ^***^, *p* ≤ 0.0001 is ^****^, ns is not significant.

## 3 Discussion

Children with acute lung injury, at risk for developing ARDS, differ in pathophysiological responses compared to adults. Ongoing postnatal alveolar and microvascular development, along with changes in chest wall compliance, anatomic dead space, and metabolic demands, place the pediatric population in unique challenges after acute lung injury. This distinction is further supported by the fact that application of biomarkers for the stratification of risk in PARDS patients required slightly lower thresholds compared to adults, highlighting the differences of these populations [28]. At the same time, survivors of PARDS carry an increased risk for long‐term complications in terms of lung function and neuro‐psychological development. Therapeutic interventions in the pediatric intensive care units, during the acute phase of the clinical syndrome, such as ventilatory support, nitric oxide use, or ECMO (extracorporeal mechanical oxygenation support) aim to sustain life and treat the symptomatology with a small effect on the underlying pathophysiology of the endothelial‐epithelial dysfunction. Novel treatments that can act to restore the epithelial/endothelial barrier and subsequently the cardiorespiratory tree are in dire need, placing the barrier's function as a prime target for therapeutic intervention. These treatments potentially may improve long‐term outcomes as well. Systemic inflammation is a major cause of lung injury in sepsis‐induced PARDS. In our study, intraperitoneal administration of LPS to neonatal mice caused significant damage to the developing lungs as shown by increased mortality, disrupted angiogenesis and alveolarization, increased capillary leakage, and increased pulmonary inflammation, all hallmarks of sepsis‐induced PARDS. Herein, we developed a novel therapeutic approach based on an LEDD nanoparticle delivery system for the potential treatment of PARDS.

We utilized a targeted NP delivery of TanFe[F], which is a small‐molecule compound that we chemically modified to minimize toxicity and to deliver the compound directly into the lung endothelial cells. Our choice of TanFe[F] was due to the fact that it is an activator of FOXF1 expression. We have previously reported that FOXF1 activation in endothelial cells is important for vascular repair and endothelial proliferation after lung injury [26]. FOXF1‐expressing endothelial progenitor cells are conserved among humans and mice and are found decreased in specific disorders of lung development [25, 29]. Increasing FOXF1 expression in the endothelium via a transgene or gene therapy improves the lung microvasculature in mouse models of bronchopulmonary dysplasia, alveolar capillary dysplasia, and idiopathic pulmonary fibrosis [15, 25, 30], making TanFe[F] an ideal pharmacological agent to restore LPS‐mediated decline in FOXF1 expression in PARDS. Using a mouse model of PARDS, we revealed that LEDD nanoparticle delivery of TanFe[F] has a direct effect on endothelial FOXF1 activation, preventing the LPS‐induced damage. The LEDD TanFe[F] treatment restores endothelial proliferation, improves capillary density and alveolarization, reduces endothelial permeability and inflammation, and increases the expression of angiogenic EC genes (Figure S15), all of which have positive effects on lung repair after neonatal lung injury. The limitation of our study is that we cannot use EC‐specific FOXF1‐deficient mice to demonstrate that the beneficial effect of TanFe[F] treatment is dependent upon FOXF1 expression in endothelial cells. Published studies reported that EC‐specific deletion of Foxf1‐floxed alleles is lethal [26].

In previous studies, we have developed a nanoparticle delivery system for lung cell‐specific delivery of DNA expression vectors [31]. However, nanoparticle delivery of drugs specifically to pulmonary endothelium has never been achieved. The nanoparticle‐mediated targeted delivery is critically important in respiratory disorders as this strategy overcomes the major barrier of nonspecific targeting seen in conventional therapeutic strategies [31]. Due to the target specificity, the new LEDD nanoparticle formulation developed in the present study potentially can be used to deliver other drugs to lung ECs in various pulmonary diseases, not only those affecting children but also neonates and adults. We confirmed the utility of the LEDD nanoparticle system to deliver drugs to pulmonary endothelium by encapsulating other small molecule compounds such as Vorinostat, RCM‐1, and TanFe. One limitation of our study is that the molecular mechanisms of lung EC selectivity of our NPs are still unknown. It is possible that lung endothelial cells have a distinct signature of glycoproteins in the negatively charged EC glycocalyx compared to EC glycocalyx signatures of other organs. A combination of charge‐dependent glycocalyx binding with selective glycoprotein recognition can be responsible for lung‐specific targeting of our NPs.

This study comes to compliment an initiative of novel therapeutic approaches in pediatric pulmonary conditions. These include the use of stem cells or their products or other biologics such as hormonal derivatives. In summary, we demonstrated that LPS injury in neonatal mice reduces the expression of FOXF1 in the pulmonary endothelium. A novel nanoparticle‐based TanFe[F] treatment strategy is effective in targeting lung endothelial cells, restoring FOXF1 expression levels in lung endothelial cells after injury. The target‐specific delivery to lung endothelial cells and promising role of FOXF1 in mediating repair and promoting angiogenesis have translational significance for treatment of pediatric lung injury and may lead to novel therapeutic strategies in pediatric ARDS.

## 4 Materials and Methods

### 4.1 Single‐Cell RNAseq Analysis

The dataset was generated using FACS‐sorted lung endothelial cells obtained from LPS‐injured mice (i.p. 12.5 mg/kg of body weight) 48 hrs after the injury. The data are deposited to GSE148499 [32]. Data analysis and visualizations were performed with Seurat V5 and R Studio 4.4.0 [33]. Briefly, separate Seurat objects were created with min.features = 200 and min.cells = 3, normalized, scaled, and variable features were found using SCT transform with default parameters [34]. RunPCA was performed using default parameters and ElbowPlot to determine the ideal principal components. To perform unsupervised clustering analysis, FindNeighbors (dims 1:20), FindClusters (resolution = 0.1), and RunUMAP (dims 1:20) were used. The cell clusters were visualized using DimPlot_scCustom, five separate cell clusters were found for the control and six clusters for the LPS group. The LPS clustering contained an artifact cluster that did not express *Pecam1* compared to the other clusters and was removed before integration. The separate Seurat objects were merged and integrated using Harmony [35]. The same processing steps as previously mentioned were performed on the integrated object.

### 4.2 Cluster and Marker Identification

To determine the identity of the integrated cell clusters, endothelial markers were utilized from published studies [36] and visualized using Dotplot_scCustom. The object was prepared for marker analysis using PrepSCTFindMarkers using default settings. To find differentially expressed markers, FindAllMarkers with logfc.threshold = 0.25, min.pct = 0.1, only positive markers, test.use = wilcox and assay = SCT was run. Ensemble IDs were converted to gene symbols in the integrated Seurat object. Five major endothelial cell types were identified gCap (*Kit, Aplnr*), aCap (*Apln, Car4*), Venous (*Eln, Ephb4*), Arterial (*Efnb2, Cxcl12*) and Lymphatic (*Prox1, Mmrn1*). Differentially expressed markers were converted from Ensembl IDs to gene symbols and filtered by p_val_adj < 0.05. The significant marker genes for gCaps and aCaps were input into Enrichrr for gene ontology analysis [37]. For gCap and aCap violin plots, ensembl IDs were converted to gene symbols in the integrated Seurat object. Select pro‐angiogenic and anti‐apoptotic genes were visualized using VlnPlot_scCustom, grouped by cluster identity and split by treatment. Box plots were added as described in published study [18]. Statistical significance for each gene was calculated using the Pairwise Wilcox Test comparing control and treatment groups, with false probability correction using the Benjamini‐Hochberg method. Means of the gene were found using GetAssayData sub‐setting based on Identity of the cells and treatment using the data plot for the corresponding Ensembl IDs of the genes of interest.

### 4.3 Animals

C57BL/6 mice were purchased from Jackson Laboratories and used for breeding to produce neonatal mice. Mice were housed in the barrier facility and used for experiments according to the protocol approved by the Animal Care and Use Committee of the University of Arizona (Protocol No. 2023‐1020). Pups obtained after breeding WT mice were challenged with 6 mg/kg of LPS at postnatal (P) days P7 and P8. The LPS dose was chosen based on previous studies reporting disrupted alveolarization and angiogenesis in mice [38]. For survival studies, 10 and 15 mg/kg doses of LPS were used. LPS was administered intraperitoneally (i.p.).

### 4.4 Generation of Nanoparticles Containing TanFe[F]

Lung endothelial cell‐specific PBAE nanoparticles were synthesized as described previously [14]. Briefly, poly‐β amino esters (PBAE) were synthesized via a two‐step Michael Addition. First, the bisphenol A ethoxylate diacrylate was mixed with 6‐amino 1‐hexanol in DMSO and incubated at 90°C for 24 hrs. Then, the mixture was added with 4’4‐trimethylenedipiperidine at 50°C for another 24 hrs. The polymer backbone was then capped with poly(ethylene glycol) amine (PEG, Mn ∼2000) and polyethylenimine (PEI, Mn ∼600). TanFe[F] was encapsulated in nanoparticles by thorough mixing and dialyzing for 24 hrs with 2–3 water changes to remove extra capping agents and free drugs. To optimize the delivery efficiency of TanFe[F], we used nanoparticles with both long (1.4DA) and short (1.6DA) backbone lengths, synthesized by controlling the diacrylate‐to‐amine (DA) ratio. Since increasing the PEG in capping agents enhances the nanoparticle's stability [31], an extra PEG group (PEG4X) was incorporated to optimize delivery efficiency by increasing the PEG percentage in the capping agents fourfold. The size and charge of nanoparticles were determined using a Malvern Zetasizer Ultra. Nanoparticles were fluorescently tagged with Cyanine 5 dye as previously reported [30, 39, 40]. The dose of TanFe[F] in nanoparticles was confirmed by using UV–vis Spectroscopy, as described in earlier studies [17]. Vorinostat, RCM‐1, and TanFe were encapsulated into nanoparticles using a similar strategy.

### 4.5 Immunostaining

Paraffin or frozen lung sections were placed on Poly L‐Lysine glass slides. Paraffin sections were stained with Hematoxyline and Eosine (H&E) for assessing the percent airspace as described [41, 42, 43, 44]. Frozen sections were stained with primary antibody against endomucin (Abcam), CD31 (BD Pharmacogen), FOXF1 (R&D System), or Ki‐67 (Abcam) followed by secondary antibody conjugated with Alexa Fluor 488 or Alexa Fluor 546 (Invitrogen) as described [39, 45, 46, 47]. The information about primary antibodies is provided in supplementary Table S1. Brightfield and Fluorescent images were taken using a Zeiss AxioImager M2 Epifluorescent microscope equipped with Axiocam digital monochrome and color cameras and Zen Pro image analysis software (Carl Zeiss Microimaging, Thornwood, NY) as described [40, 45, 48, 49]. All images were analyzed using ImageJ to quantify the percent airspace, endomucin area, or percentages of FOXF1^+^ and Ki‐67^+^ endothelial cells as described [50, 51].

### 4.6 QRT‐PCR

Total RNA was prepared from lung tissue using Trizol (Applied Biosystem, Waltham, MA) and reverse transcribed to synthesize cDNA using the High‐Capacity cDNA Synthesis Kit (Applied Biosystem, Waltham, MA). cDNA was amplified using gene‐specific probes and the Custom TaqMan Gene Expression Assay (Applied Biosystem, Waltham, MA) as described [47, 48, 52, 53]. The following TaqMan probes were used for amplification: *Actb: Mm00667939_s1, Tnfα: Mm0443259_m1, Il1b: Mm00434228_m1, Erg: Mm01214244_m1, Tmem100: Mm00471353_m1*. Relative expression of each gene was determined by the Delta2Ct method and normalized to beta‐actin mRNA.

### 4.7 Western Blot and Dual HiBiT Assay

For western blot, 20 µg of protein was loaded in Mini‐protean TGX precast gels (Bio‐Rad). Proteins were transferred to Nitrocellulose membrane and blots were blocked using 3% BSA and incubated with anti‐FOXF1 or anti‐Vinculin primary antibodies followed by HRP‐conjugated secondary antibodies. FOXF1 expression was normalized to vinculin to quantify the FOXF1 protein levels as described [9, 47]. For Nano‐Glo HiBiT assay, human *FOXF1* cDNA was cloned into the pBit3.1‐C vector (*FOXF1‐pBit* plasmid) to produce HiBiT‐tagged human *FOXF1* construct. Mouse fetal lung endothelial MFLM‐91U cells were cultured in 96‐well plates and transfected with *FOXF1‐pBit* plasmid using TransIT‐X2 (Mirus Bio). 10 or 20 µm of TanFe[F] diluted in DMSO was added to the MFLM‐91U cells transfected with *FOXF1‐pBit* plasmid for 24 hrs. HiBiT‐tagged FOXF1 protein levels were then measured using the Nano‐Glo HiBiT Lytic detection system (Promega) 24 hrs after TanFe[F] treatment. For this purpose, 40 µl of PBS and 40 µl of HiBiT Lytic reagent (40 µl HiBiT Lytic Buffer, 0.4 µl LgBiT protein, 0.8 µl Nano‐Glo HiBiT Lytic substrate) were added to each well of MFLM‐91U cells. The cell culture plate was then placed on an orbital shaker for 10 min at 300–600 rpm, and bioluminescence was measured using a Cytation 1 microplate reader (BioTek). For in vivo assay, 1 mg of *FOXF1‐HiBiT* construct was encapsulated into PBAE‐P22‐F1 nanoparticles [14] and injected in mice through retro‐orbital route. Twenty‐four hours after the plasmid delivery, mice were given the TanFe[F] encapsulated in PBAE‐1.4DA‐PEG4X nanoparticles and harvested after 24 hrs to quantify relative luminescence in lung single‐cell suspension.

### 4.8 Evans Blue Permeability Assay

Endothelial permeability was analyzed by measuring extravasated Evans blue dye in perfused lung tissue. Evans blue was injected 4 hrs prior to the lung harvest. Lungs were perfused with PBS at the time of harvest to remove the intravascular Evans blue dye. Harvested lungs were incubated in 2 mL of formamide, and extravasated Evans blue was quantified by absorbance using formamide as blank.

### 4.9 Total Protein, LDH Assay and Differential Counting

Total protein in BAL fluid (BALF) was quantified using the Pierce total protein assay kit (Invitrogen, Waltham, MA). LDH activity in BALF was measured using the CYQUANT LDH Cytotoxicity Assay kit (Invitrogen, Waltham, MA). For differential counting, BALF was collected on Poly L‐Lysine glass slide using cytospin centrifugation. The slides were fixed and quick stained using the Differential Quick III Stain Kit (Polysciences). Slides were imaged using an EVOS7000 bright‐field microscope to quantify the percentage of macrophages and neutrophils.

### 4.10 Flow Cytometry

Flow cytometry was performed on enzymatically digested lung cell suspensions using a 4‐laser 14‐color Attune NxT Flow Cytometer with CytKick Max Autosampler at the flow cytometry core at the University of Arizona College of Medicine‐Phoenix. Following antibodies were used to identify the hematopoietic cells (CD31^−^ CD45^+^), endothelial cells (CD31^+^CD45^−^), endothelial progenitor cells (CD45^−^CD31^+^cKIT^+^), fibroblasts (CD140a^+^CD31^−^CD45^−^), epithelial cells (CD326^+^CD31^−^CD45^−^CD140a^−^) and pericytes (NG2^+^CD326^−^CD31^−^CD45^−^CD140a^−^): anti‐CD45 (clone30‐F11; Biolegend), anti‐CD31 (clone MEC13.3; eBioscience), anti‐cKIT (clone 2B8; Biolegend), anti‐CD326 (clone G8.8; Biolegend), anti‐CD140a (clone APA5; eBioscience), anti‐NG2 (Millipore). Dead cells were identified using 7‐AAD Fixable Viability dye (Biolegend) as described [25].

### 4.11 Statistical Analysis

Statistical analyses were performed using One‐Way ANOVA, Two‐Way ANOVA (with Tukey's post hoc multiple comparison test), or Welch t‐test using GraphPad Prism V8 (GraphPad, La Jolla, CA, USA). Results were reported as mean ± standard deviation (SD). P ≤ 0.05 was considered as statistically significant.

## Author Contributions

Conceptualization: ANM and VVK. Methodology: ANM, ZD, AM, AK, MO, FK, JD, EL, Y‐W L, AD. Formal analysis: ANM, AM, AK, MO, JD, ZD, EL, Y‐W L, HB, AD. Visualization: ANM, ZD, JD, FK, AD. Funding acquisition: VVK and TVK. Writing – original draft: ANM and VVK. Writing – review & editing: VVK, ANM, AD.

## Funding

This work is supported by National Heart, Lung, and Blood Institute grants HL141174, HL149631, and HL152973 to VVK.

## Consent

The authors have nothing to report applicable.

## Conflicts of Interest

The authors declare no conflicts of interest.

## Supporting information




**Supporting File**: advs78292‐sup‐0001‐SuppMat.pdf.

## Data Availability

The data that support the findings of this study are available from the corresponding author upon reasonable request.
