# Supplementary material for: Development of Versatile Nanoparticle System for Efficient Drug Delivery into Pulmonary Endothelium
Source: Adv Sci (Weinh). 2026 Oct 14:e78292. Online ahead of print. doi: 10.1002/advs.78292 (PMC13669217; doi:10.1002/advs.78292)
Supplement: Supplementary file 1 [file ADVS-9999-e78292-s001.pdf]

Supplementary Materials

Development of Versatile Nanoparticle System for Efficient Drug Delivery into Pulmonary Endothelium

Afzaal N. Mohammed[1‡], Zicheng Deng[1‡], Arnab Misra[1], Marcus Orzabal[1], Ananya Kondapally[1], Enhong Li[1], Jonathan Do[1], Ying-Wei Lan[1], Fatemeh Kohram[1], Hunter Blackburn[1], Andreas Damianos[1, 2], Tanya V. Kalin[1,3], Vladimir V. Kalinichenko*[1,2]

[1]Phoenix Children's Research Institute, Department of Child Health, University of Arizona College of Medicine-Phoenix, Phoenix, AZ 85004, USA.

[2]Division of Neonatology, Phoenix Children's Hospital, Phoenix, AZ 85004, USA.

[3]Center for Cancer and Blood Diseases, Phoenix Children's Hospital, Phoenix, AZ 85004, USA.

Supplementary Methods

Nanoparticle characterization: Nanoparticles (NPs) were characterized for their encapsulation efficiency, release profile, serum stability, and uptake by the endothelial cells. Encapsulation was determined by the absorbance of nanoparticle -TanFe[F] complex at 365 nm wavelength which is specific for TanFe[F]. Release profile was determined by incubating nanoparticles in analysis tube (Thermo Fisher Scientific Inc, Waltham, MA) and taking the absorbance reading each hour over the period of 24 hrs at 37°C. Size and charge of different NP formulations were determined using Zetasizer Ultra (Malvern Panalytical Ltd, Malvern, UK). To demonstrate the uptake of nanoparticles by endothelial cells, nanoparticles were tagged with Cyanine 5 dye and added to mouse endothelial MFLM-91U cells grown on chamber slides. Four hours after exposure, cells were fixed and stained with a primary antibody against Occludin (Proteintech, Rosemont, IL), followed by an Alexa Fluor 647-conjugated secondary antibody. Fluorescence images were acquired using a ZEISS LSM 800 Airyscan laser-scanning confocal microscope. Images were processed and analyzed using ZEN Pro image analysis software.

qRT-PCR: Human HPMEC cells were cultured in complete endothelial growth medium under standard culture conditions. Cells were seeded into 6-well plates and stimulated with lipopolysaccharide (LPS; 1 μg/mL) for 24 hrs, followed by treatment with either TanFe[F] or vehicle (DMSO) for an additional 24 hrs. Total RNA was isolated using TRIzol Reagent (Applied Biosystems, Waltham, MA), and cDNA was synthesized using the High Capacity cDNA Reverse Transcription Kit (Applied Biosystems, Waltham, MA). cDNA was amplified using gene specific probes and custom TaqMan gene expression assays (Applied Biosystems, Waltham, MA). The following TaqMan assays were used *ACTB:* (Hs01060665_g1), *S1PR1* (Hs01922614_s1), *APLNR* (Hs00270873_s1), and *IL1B* (Hs01555411_g1).

Immunoprecipitation: Immunoprecipitation of FOXF1 protein was performed using MFLM-91U cells exposed to 20 μM of TanFe[F] or vehicle control (DMSO) for 24 hrs. Following treatment, cells were harvested to extract total protein. Co-IP was conducted using the Dynabeads Co-Immunoprecipitation Kit (Thermo Fisher Scientific, Waltham, MA) according to the manufacturer's instructions. Briefly, anti-FOXF1 antibody was covalently coupled to Dynabeads M-270 Epoxy beads on a roller at 37°C for 24 hrs. The antibody-bead complexes were washed using a magnetic separation rack (DynaMag-2) and incubated with total protein lysates at 4°C for 30 min. Target protein complexes were eluted using the kit elution buffer via magnetic separation followed by Western blot for HECTD1 protein.

Supplementary Table S1: List of antibodies and dilutions.

Antibody	Company	Cat#	Dilutions	Assay
CD31	eBiosciences	48-0311-82	1:50	Flow Cytometry
CD45	Biolegend	103128	1:100	Flow Cytometry
CD117	Biolegend	105835	1:100	Flow Cytometry
CD140a	eBioscience	25-1401-82	1:100	Flow Cytometry
CD326	Biolegend	118205	1:100	Flow Cytometry
NG2	Millipore	AB5320C3	1:100	Flow Cytometry
FOXF1	R &D	AF4798	1:1000	Western
Vinculin	Cell Signaling	13901S	1:1000	Western
HECTD1	Novus Biologicals	NBP-149925	1:1000	Western
Endomucin	Abcam	ab106100	1:100	Immunofluorescence
CD31	BD Biosciences	553370	1:100	Immunofluorescence
FOXF1	R & D	AF4798	1:100	Immunofluorescence
Ki-67	Abcam	ab16667	1:100	Immunofluorescence
Occludin	Proteintech	27260-1AP	1:250	Immunofluorescence
Secondary	Invitrogen	A-11006	1:200	Immunofluorescence
Secondary	Invitrogen	A-11056	1:200	Immunofluorescence
Seconday	Invitrogen	A-11035	1:200	Immunfluoroscence

Supplementary Figures

Figure S1

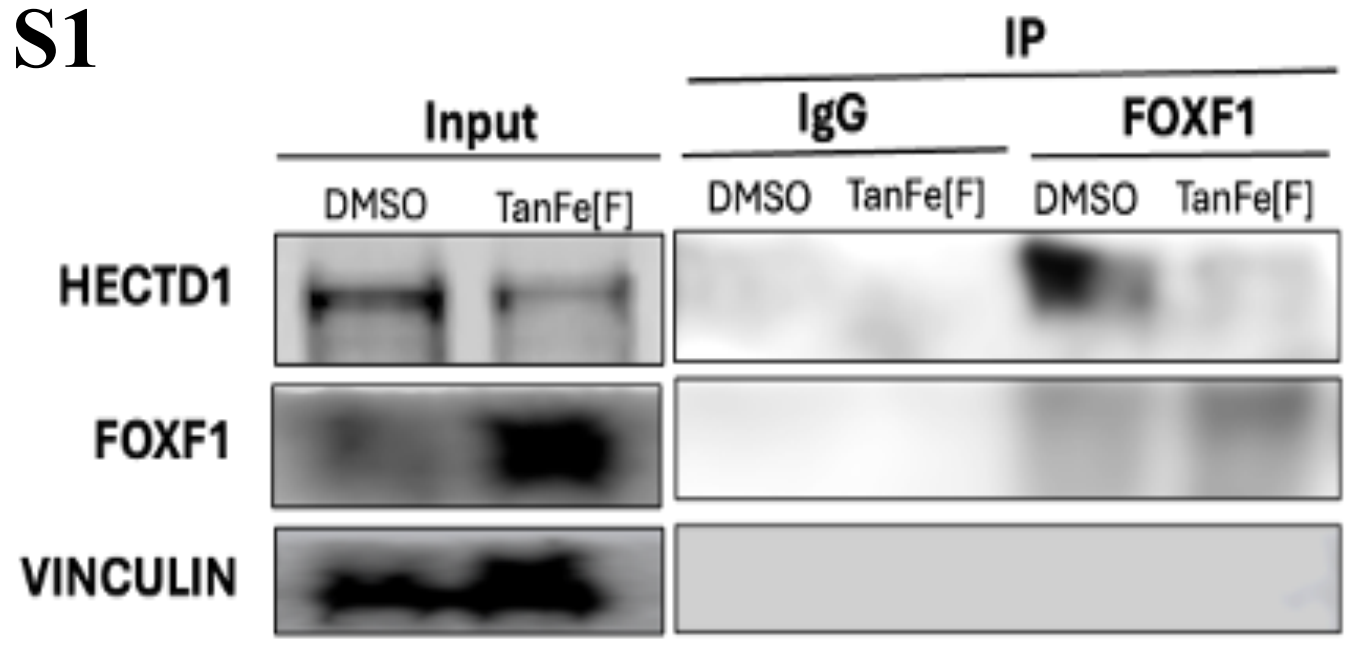

Figure S1: TanFe[F] decreases protein-protein interaction between FOXF1 and HECTD1. MFLM-91U cells were treated with 20 µM of TanFe[F] or vehicle control (DMSO). 24 hrs following exposure, cell were harvested, immunoprecipitated using anti-FOXF1 antibody, and analyzed by Western blot alongside input samples to evaluate HECTD1 and FOXF1 protein levels.

Figure S2

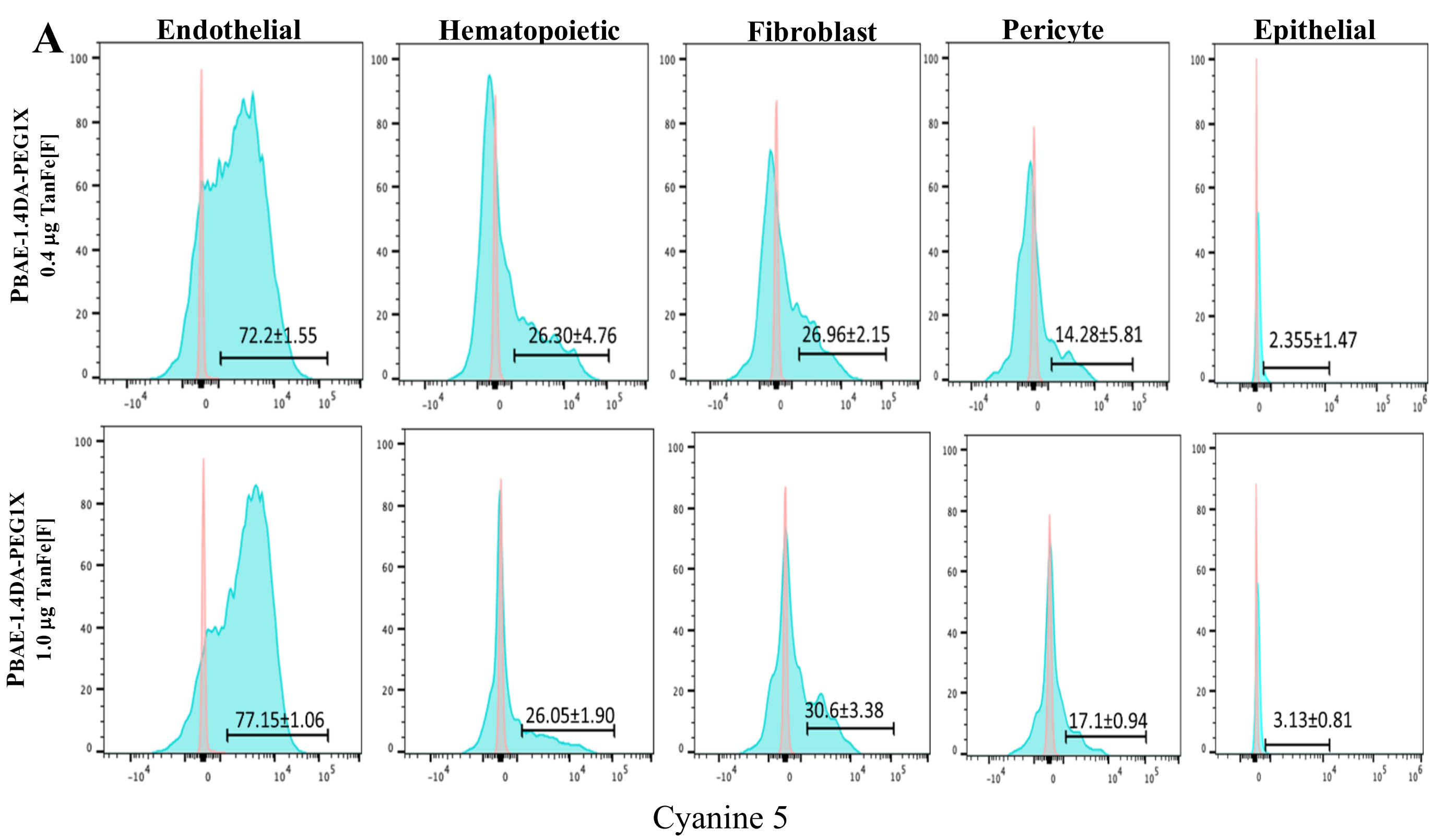

B

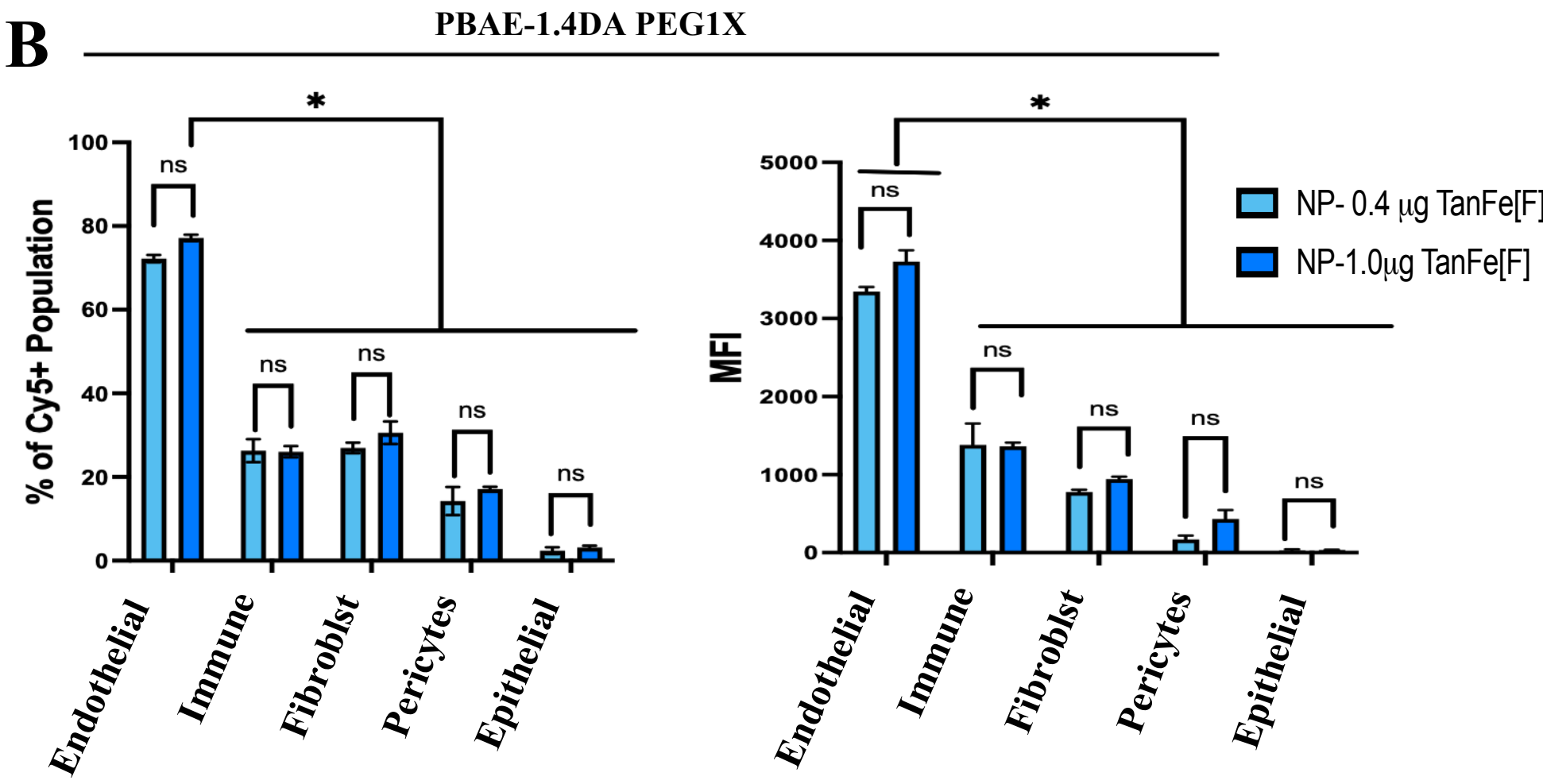

Figure S2. TanFe[F] can be encapsulated into PBAE-1.4DA-PEG1X nanoparticles and delivered to pulmonary cells *in vivo*: 0.4 or 1 μg of TanFe[F] compound encapsulated into PBAE-1.4DA-PEG1X nanoparticles labelled with Cyanine 5 (Cy5). The hydrophobicity of the NPs was controlled by the length of the hydrophobic backbone (long backbone: 1.4DA). Nanoparticle-encapsulated TanFe[F] was delivered to C57BL6 mice using a retro-orbital injection. **(A)** Flow cytometry analysis of NP distribution in different pulmonary cell types from uninjected mice (red curve) or mice injected with TanFe[F] encapsulated into PBAE-1.4 DA-PEG1X formulation of nanoparticles (blue curve). **(B)** Mean fluorescent intensity (MFI) and percentage of cells receiving the nanoparticle-TanFe[F] complexes. Statistical significance was calculated using one-way ANOVA with Tukey's posthoc multiple comparisons. Data show mean ± SD, P≤0.05 is shown with *, ns is not significant.

Figure S3

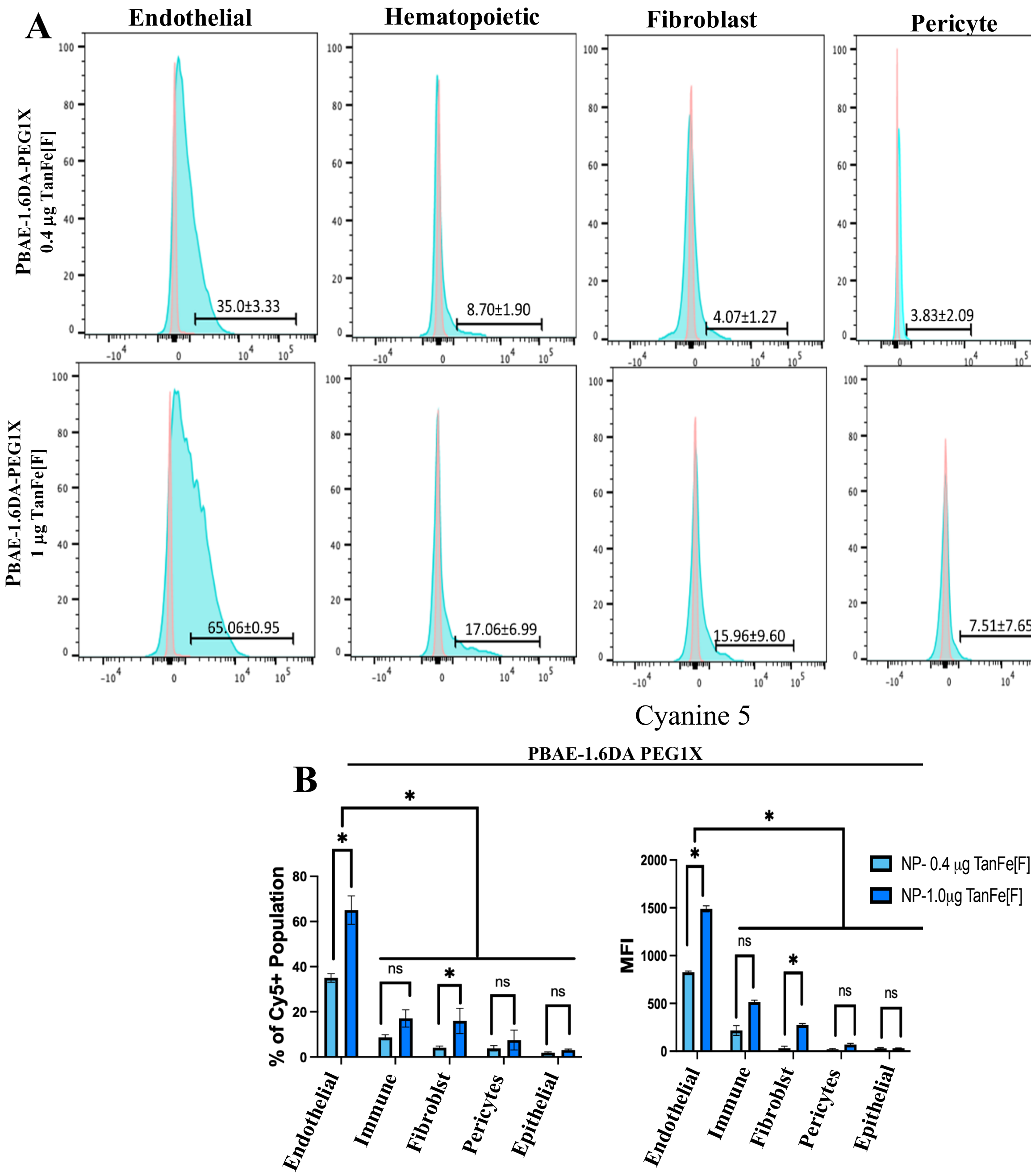
A
Endothelial
Hematopoietic
Fibroblast
Pericyte
Epithelial
PBAE-1.6DA-PEG1X 0.4 µg TanFe[F]
PBAE-1.6DA-PEG1X 1 µg TanFe[F]
35.0±3.33
8.70±1.90
4.07±1.27
3.83±2.09
1.86±0.67
65.06±0.95
17.06±6.99
15.96±9.60
7.51±7.65
3.05±0.73
Cyanine 5
B
PBAE-1.6DA PEG1X
% of Cy5+ Population
MFI
NP- 0.4 µg TanFe[F]
NP-1.0µg TanFe[F]
Endothelial
Immune
Fibroblst
Pericytes
Epithelial
ns
*

Figure S3. TanFe[F] can be encapsulated into PBAE-1.6DA-PEG1X nanoparticles for *in vivo* lung delivery. 0.4 or 1 μg of TanFe[F] compound encapsulated into PBAE-1.6DA-PEG1X labelled with Cyanine 5 (Cy5). The hydrophobicity of the NPs was controlled by length of the hydrophobic backbone (short backbone: 1.6DA). Nanoparticles with TanFe[F] were delivered into the C57BL6 mice using a retro-orbital injection. **(A)** Flow cytometry analysis of NP distribution in different pulmonary cell types of uninjected mice (red curve) and mice injected with TanFe[F] encapsulated into PBAE-1.6DA-PEG1X nanoparticles (blue curve) **(B)** Mean fluorescent intensity (MFI) and percentage of cells receiving the nanoparticle-TanFe[F] comlexes. Statistical significance was calculated using one-way ANOVA with Tukey's posthoc multiple comparisons. Data show mean ± SD , $P \leq 0.05$ *, ns is not significant.

Figure S4

A. 0.4 μg TanFe[F] encapsulated in PBAE nanoparticles

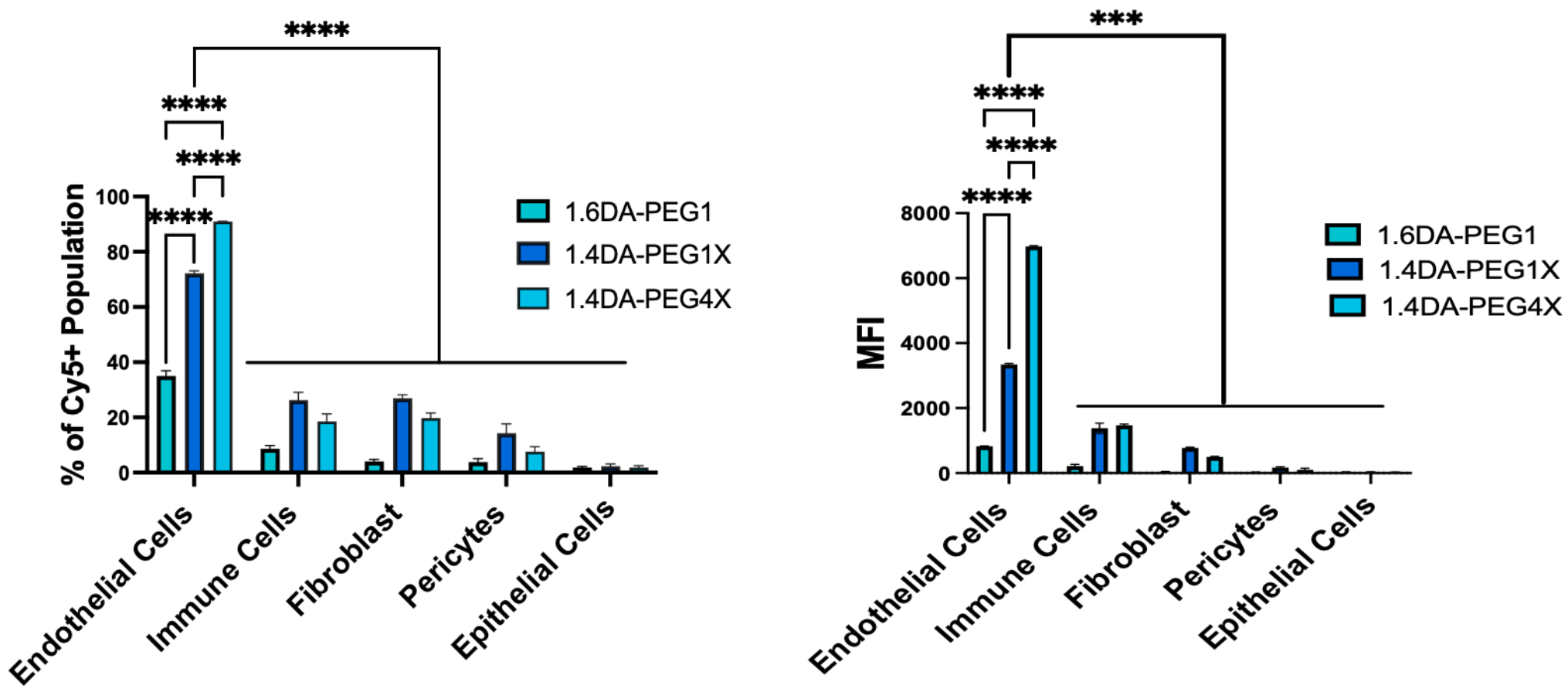

B. 1.0 μg TanFe[F] encapsulated in PBAE nanoparticles

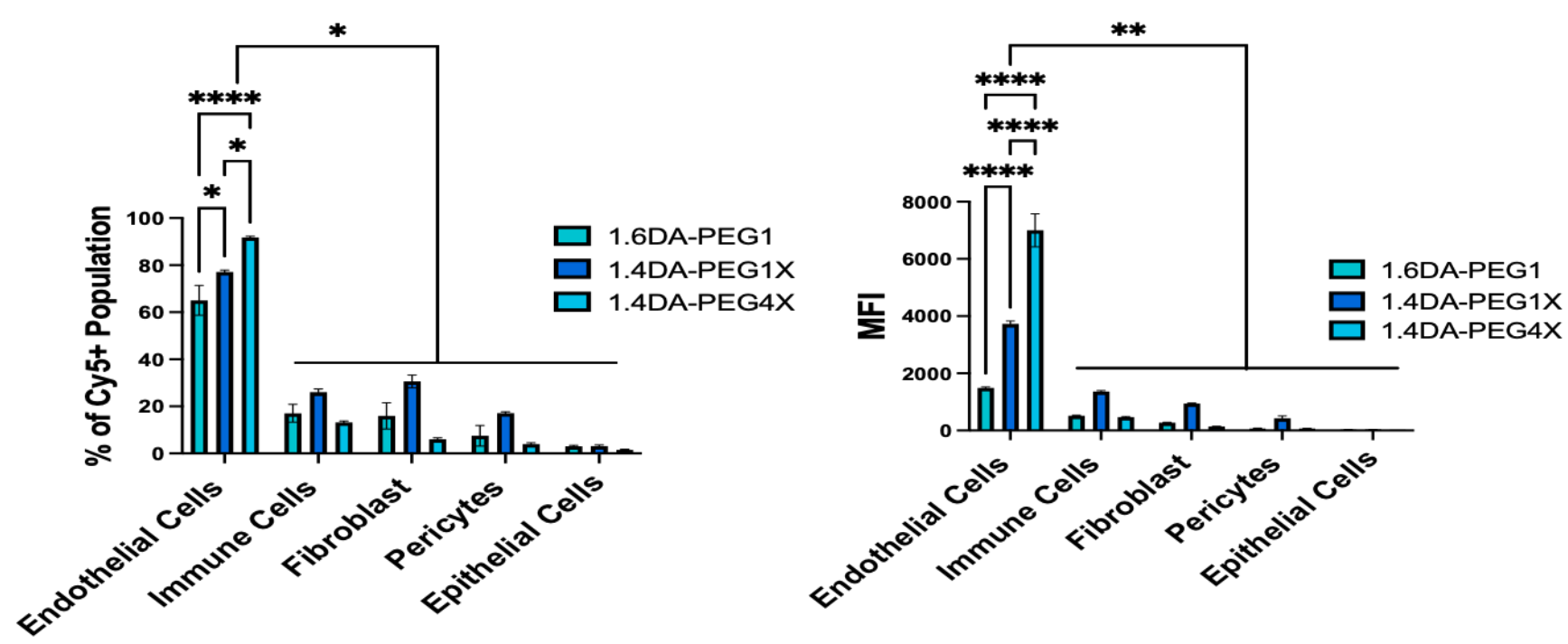

C. Size and charge of different formulations of nanoparticles

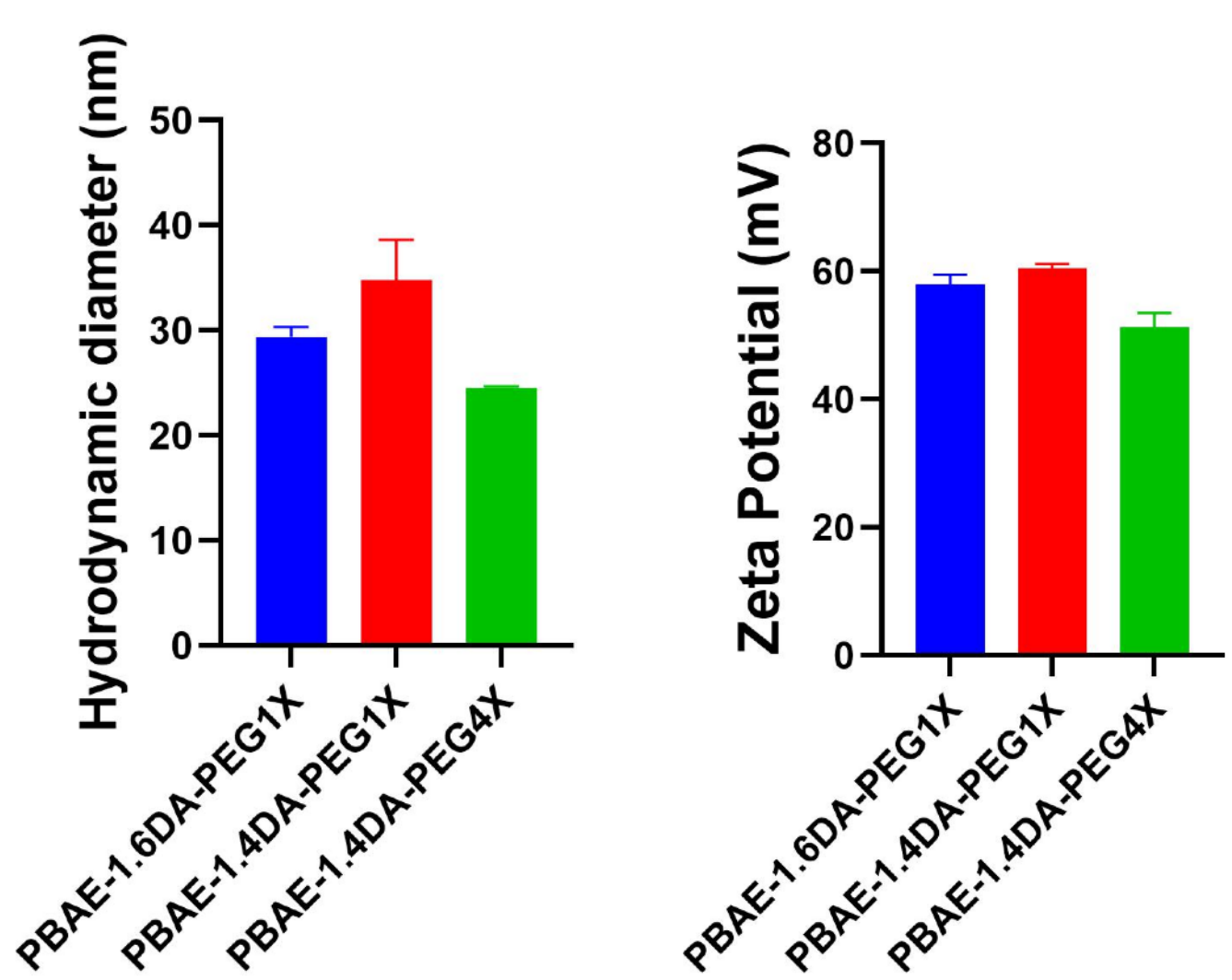

Figure S4. **TanFe[F] encapsulated into PBAE-1.4DA-PEG4X exhibit greater lung endothelial targeting compared to other formulations**. 0.4 or 1 µg of the TanFe[F] compound was encapsulated into PBAE-1.4DA-PEG4X, PBAE-1.4DA-PEG1X, or PBAE-1.6DA-PEG1X nanoparticles labelled with Cyanine 5 (Cy5). **(A-B)** The percentage of $Cy5^+$ cells and Mean Fluorescent Intensity (MFI) indicate PBAE-1.4DA-PEG4X formulation exhibits higher efficiency for targeting lung endothelial cells. Statistical significance was calculated using two-way ANOVA with Tukey's posthoc multiple comparisons. **(C)** Hydrodynamic diameter and zeta potential of PBAE-1.4DA-PEG4X formulation was comparable with to other fomulations. Data show mean ± SD, n= 3/group. P≤0.05 is *, P≤0.01 is **, P≤0.001 is ***, P≤0.0001 is ****, ns is not significant.

Figure S5

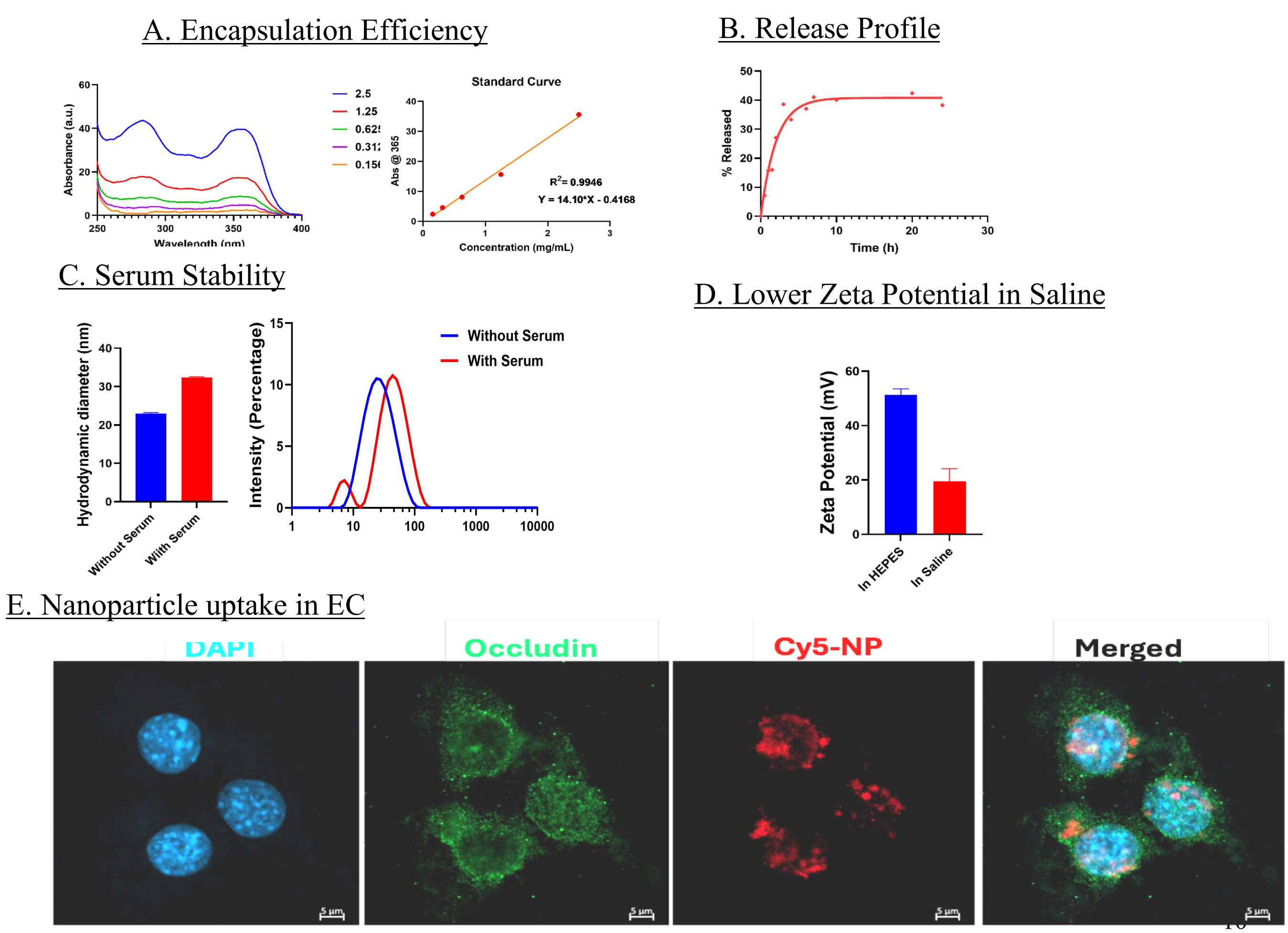

Figure S5: **Characterization and internalization of PBAE-1.4DA-PEG4X nanoparticles. (A)** Encapsulation efficiency of PBAE-1.4DA-PEG4X nanoparticles was determined by UV-Vis absorbance. The data indicate a characteristic peak at a wavelength of 365 nm. **(B)** The release profile indicates that the maximum release was achieved at the six-hour time point.

(C) PBAE-1.4DA PEG4X nanoparticles are stable in serum as indicated by stable hydrodynamic diameter and size distribution. **(D)** Zeta potential of nanoparticles depends upon PH and ionic strength of solution as indicated by lower zeta potential in saline as compared to HEPES buffer **(E)** MFLM-91U cells were exposed to PBAE-1.4DA-PEG4X nanoparticles labelled with Cyanine 5 (Cy5) dye. Four hours following the exposure, cells were stained with antibody against Occludin followed by secondary antibody conjugated with Alexa Fluor 647. Fluorescence images were obtained using at Zeiss LSM-800 Airyscan Confocal Microscope. Scale bars are 5 microns. Data indicate efficient uptake of nanoparticles by endothelial cells.

Figure S6

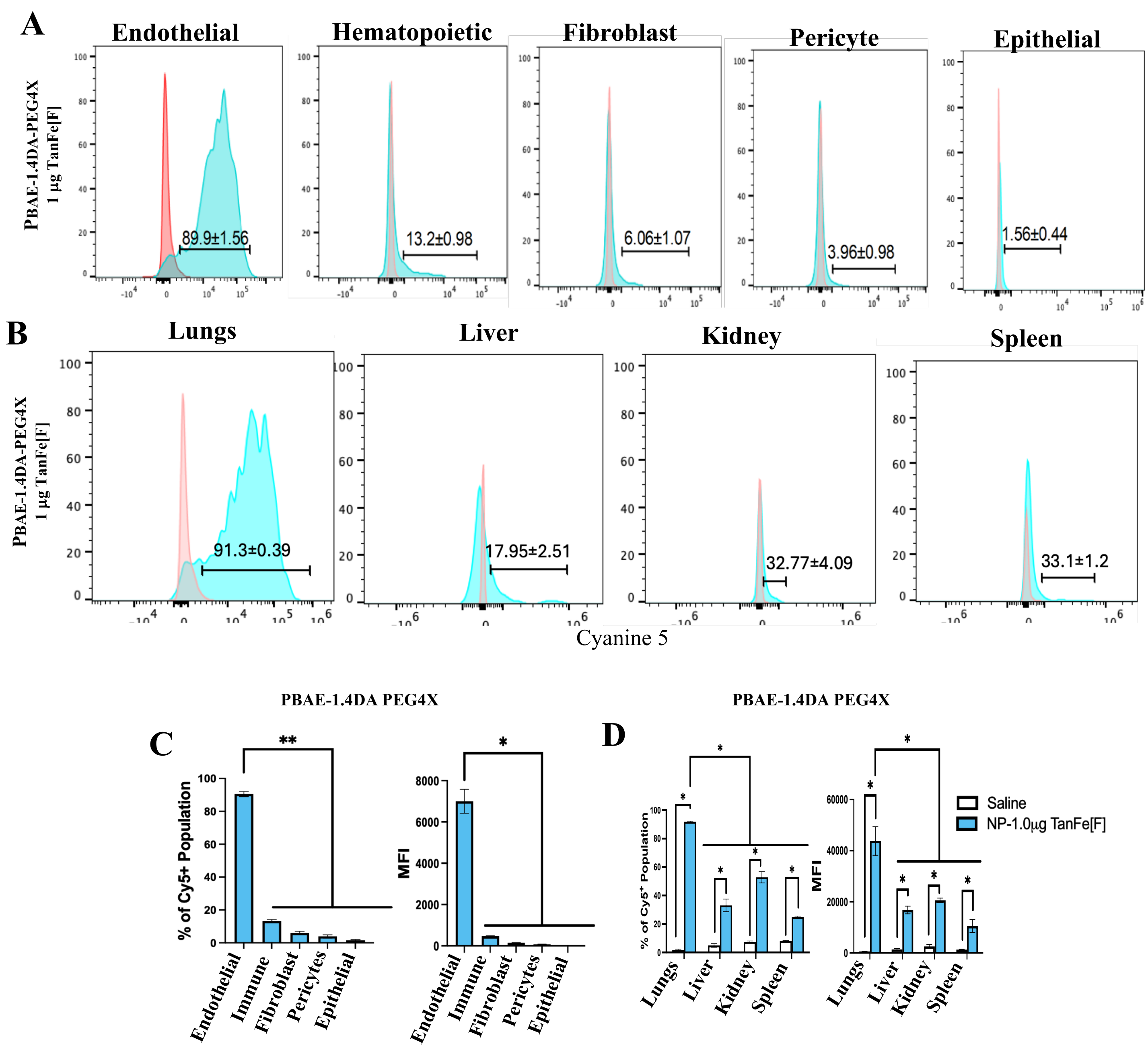

Figure S6. **TanFe[F] encapsulated into PBAE-1.4DA-PEG4X nanoparticles exhibit greater endothelial targeting compared to ECs from other organs**. 1 µg of TanFe[F] compound was encapsulated into PBAE-1.4DA-PEG4X nanoparticles labelled with Cyanine 5 (Cy5). The hydrophobicity of NPs was controlled by length of the hydrophobic backbone (long backbone: 1.4DA). NP-TanFe[F] complexes were delivered to C57BL6 mice lungs using retro-orbital

injection. **(A)** Flow cytometry analysis of NP distribution in different pulmonary cell types or **(B)** ECs from different organs from uninjected mice (red curve) or mice injected with nanoparticle-TanFe[F] complexes (blue curve) **(C)** The percentage of $Cy5^+$ cells and Mean Fluorescent Intensity (MFI) are shown confirming the EC-selective delivery of NPs. **(D)** The percentage of $Cy5^+$ ECs and MFI in ECs of different organs are shown. Statistical significance was calculated using one-way ANOVA with Tukey's posthoc multiple comparisons. Data show mean ± SD, n= 3 mice per group. P≤0.05 is *, P≤0.01 is **, ns is not significant.

Figure S7

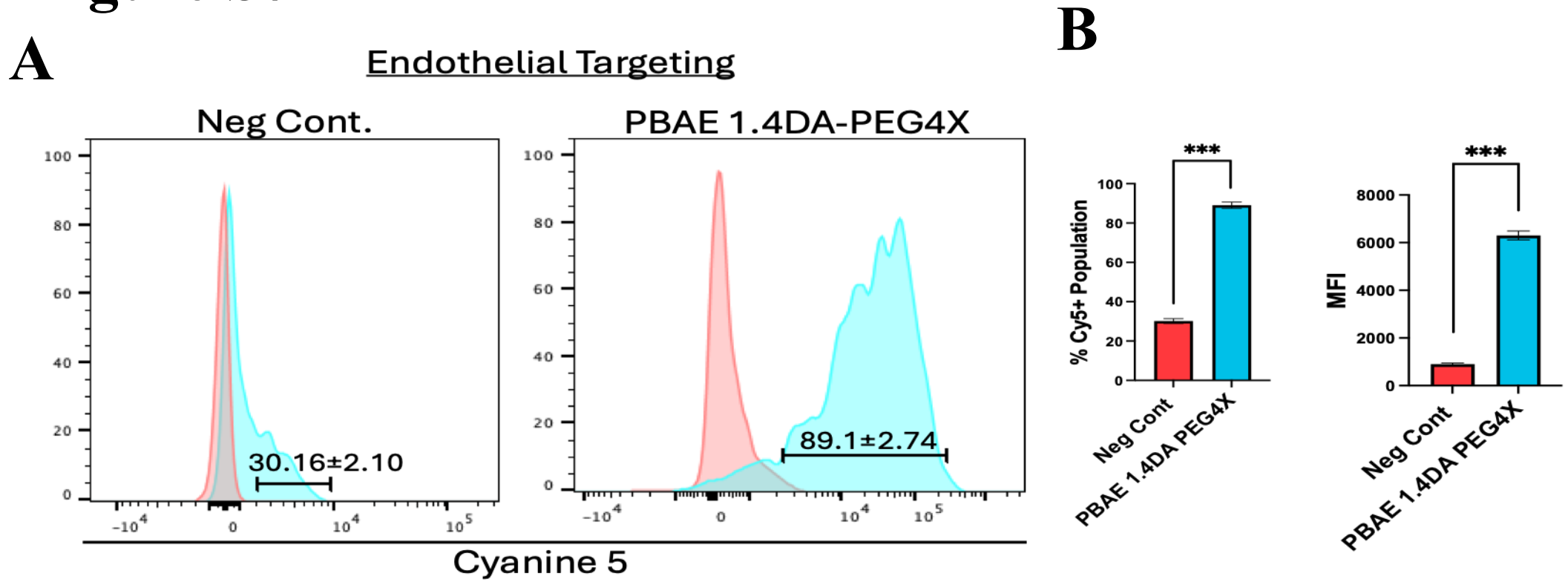

Figure S7. **The use of negative control nanoparticles generated by lowering the PEG content and fewer backbones**. Negative control nanoparticles were designed by lowering the PEG content and using fewer backbones. 0.4 µg of the TanFe[F] compound was encapsulated into either negative control NPs, or PBAE-1.4DA-PEG4X nanoparticles labelled with Cyanine 5 (Cy5). NP-TanFe[F] complexes were delivered to C57BL6 mice using retro-orbital injection. **(A)** Flow cytometry analysis of lung endothelial cells from uninjected mice (red curve) or mice injected with nanoparticle-TanFe[F] complexes (blue curve) **(B)** The percentage of $Cy5^+$ cells and Mean Fluorescent Intensity (MFI) indicate lower EC targeting by negative control NP. Statistical significance was calculated using Welch t-test. Data show mean ± SD, n= 3 mice per group. P≤0.001 is ***.

Figure S8

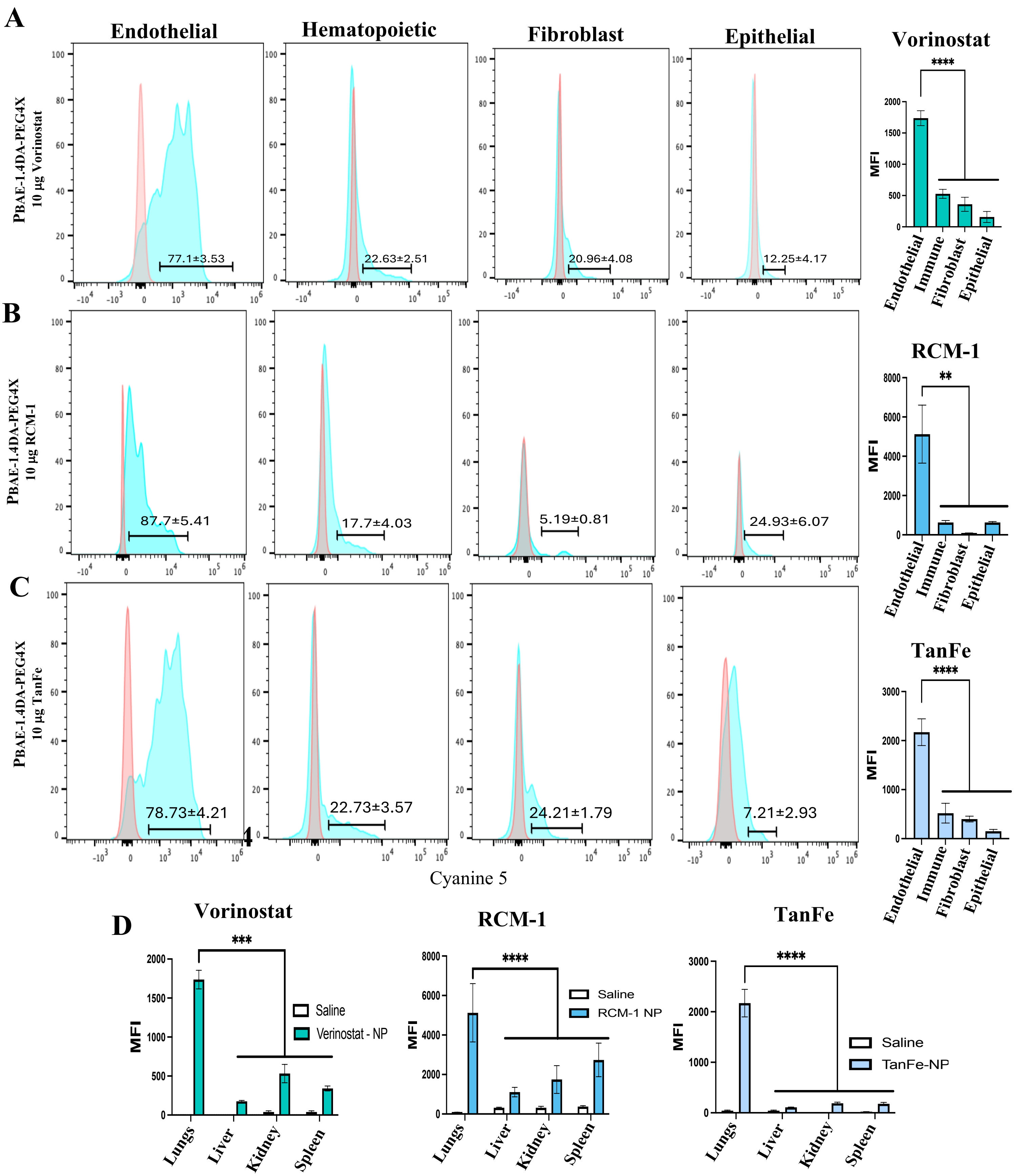
A
B
C
D
Endothelial
Hematopoietic
Fibroblast
Epithelial
PBAE-1.4DA-PEG4X 10 µg Vorinostat
PBAE-1.4DA-PEG4X 10 µg RCM-1
PBAE-1.4DA-PEG4X 10 µg TanFe
77.1±3.53
22.63±2.51
20.96±4.08
12.25±4.17
87.7±5.41
17.7±4.03
5.19±0.81
24.93±6.07
78.73±4.21
22.73±3.57
24.21±1.79
7.21±2.93
Cyanine 5
Vorinostat
RCM-1
TanFe
MFI
****
**
***
Endothelial
Immune
Fibroblast
Epithelial
Saline
Verinostat - NP
RCM-1 NP
TanFe-NP
Lungs
Liver
Kidney
Spleen

Figure S8 : PBAE-1.4DA-PEG4X nanoparticles deliver Vorinostat, RCM-1, and TanFe to lung ECs. 10 μg of Vorinostat, RCM-1, or TanFe compounds were encapsulated into PBAE-1.4DA-PEG4X nanoparticles labelled with Cyanine 5 (Cy5). **(A)** Flow cytometry analysis of NP distribution in different pulmonary cell types from uninjected mice (red curve) or mice injected with nanoparticle-Vorinostat complexes (blue curve). The percentage of $Cy5^+$ cells and Mean Fluorescent Intensity (MFI) show potential delivery of the compound to lung ECs. **(B)** Flow cytometry analysis of NP distribution in different pulmonary cell types from uninjected mice (red curve) or mice injected with nanoparticle-RCM-1 complexes (blue curve). The percentage of $Cy5^+$ cells and MFI show EC-selective delivery of NPs. **(C)** Flow cytometry analysis of NP distribution in different pulmonary cell types from uninjected mice (red curve) or mice injected with nanoparticle-TanFe complexes (blue curve). The percentage of $Cy5^+$ cells and MFI show EC-selective delivery of NPs. **(D)** Flow cytometry analysis show MFI in ECs of different organs from mice injected with nanoparticle-Vorinostat, nanoparticle-RCM-1, or nanoparticle-TanFe complexes. Statistical significance was calculated using one-way ANOVA with Tukey's posthoc multiple comparisons. Data show mean ± SD, n= 3 mice per group. $P \leq 0.01$ is **, $P \leq 0.001$ is ***, $P \leq 0.0001$ ****.

Figure S9

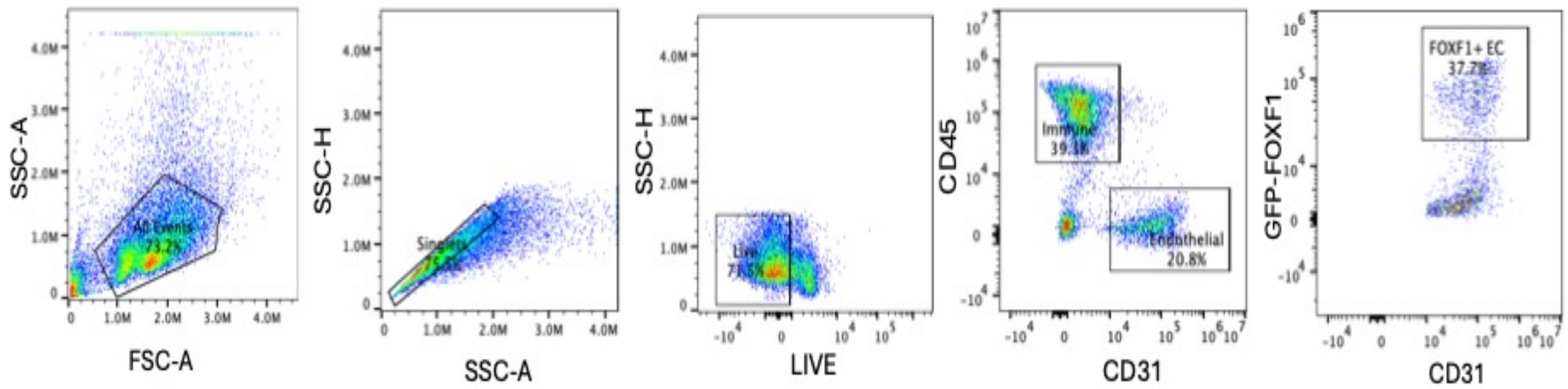

Figure S9. Flow cytometry was performed to identify FOXF1^{+} endothelial cells.

Representative gating strategy to identify FOXF1^{+} endothelial cells in transgenic reporter mice expressing green fluorescence protein (GFP) under the control of the FOXF1 promoter.

Figure S10

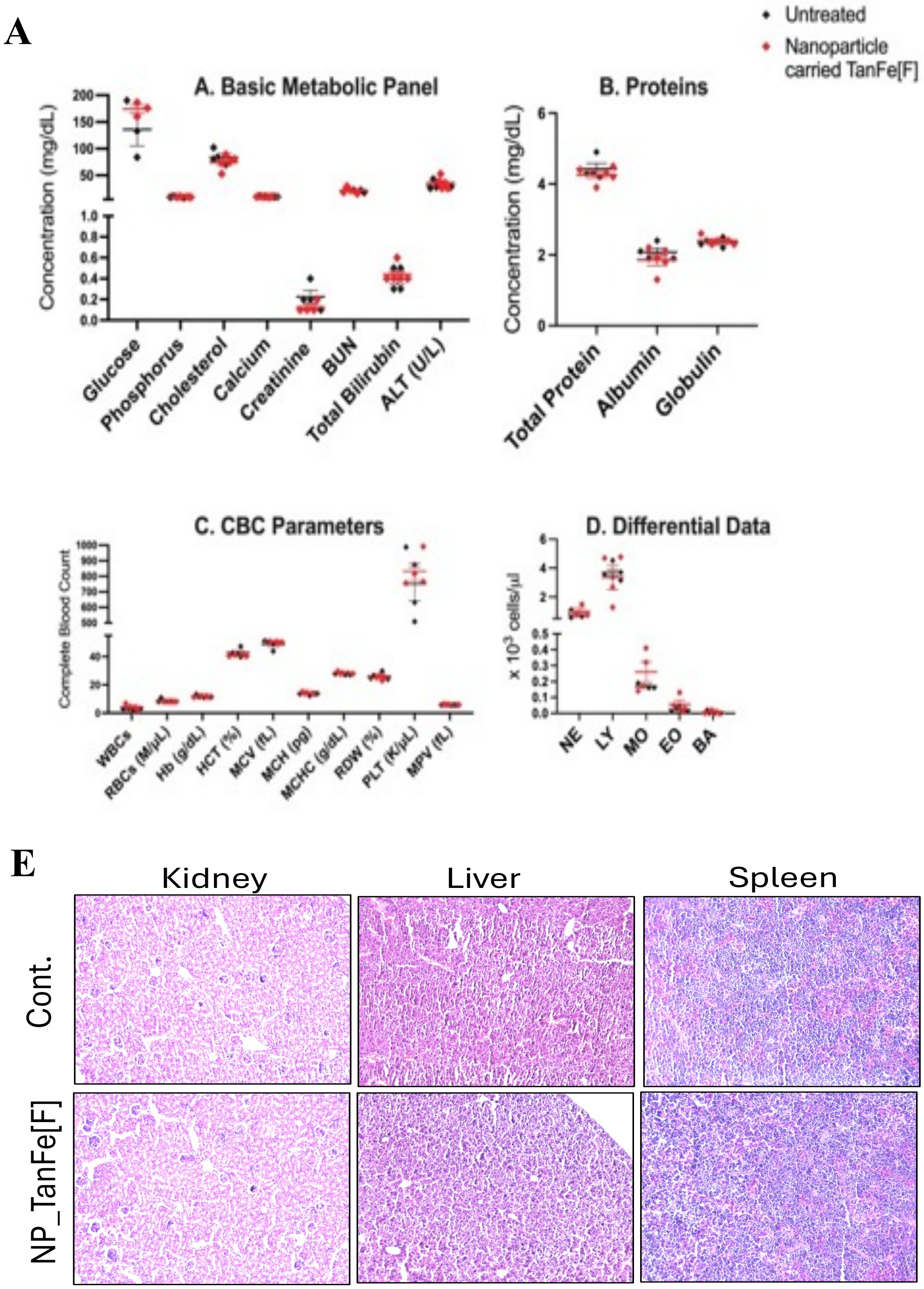
A
Untreated
Nanoparticle carried TanFe[F]
A. Basic Metabolic Panel
Concentration (mg/dL)
Glucose
Phosphorus
Cholesterol
Calcium
Creatinine
BUN
Total Bilirubin
ALT (U/L)
B. Proteins
Concentration (mg/dL)
Total Protein
Albumin
Globulin
C. CBC Parameters
Complete Blood Count
WBCs
RBCs (M/μL)
Hb (g/dL)
HCT (%)
MCV (fL)
MCH (pg)
MCHC (g/dL)
RDW (%)
PLT (K/μL)
MPV (fL)
D. Differential Data
x 10³ cells/μl
NE
LY
MO
EO
BA
E
Kidney
Liver
Spleen
Cont.
NP_TanFe[F]

Figure S10. Toxicity parameters in the peripheral blood and serum of mice treated with nanoparticle-TanFe[F] complexes. Mice treated with 0.4 μg of TanFe[F] encapsulated into PBAE-1.4DA-PEG4X nanoparticles was delivered i.v. on P13 and P14. Mice were harvested on P15 to collect the blood, serum, and organs for histopathology. **(A-D)** Toxicity parameters in blood and serum samples indicate no significant toxicological outcomes in mice injected twice with nanoparticle-encapsulated TanFe[F]. Data show mean ± SD, n= 3-5 mice per group. **(E)** Hematoxylin & Eosin staining in kidney, liver, and spleen indicate no signs of toxicity in nanoparticle-treated mice. Abbreviations- BUN: blood urea nitrogen; ALT: alanine aminotransferase; WBCs: white blood cells; RBCs: red blood cells; Hb: hemoglobin; HCT: hematocrit; MCV: mean corpuscular volume; MCH: mean corpuscular hemoglobin; MCHC: mean corpuscular hemoglobin concentration; RDW: red cell distribution width; PLT: platelet count; MPV: mean platelet volume; NE: neutrophils; LY: lymphocytes; MO: monocytes; EO: eosinophils; BA: basophils.

Figure S11

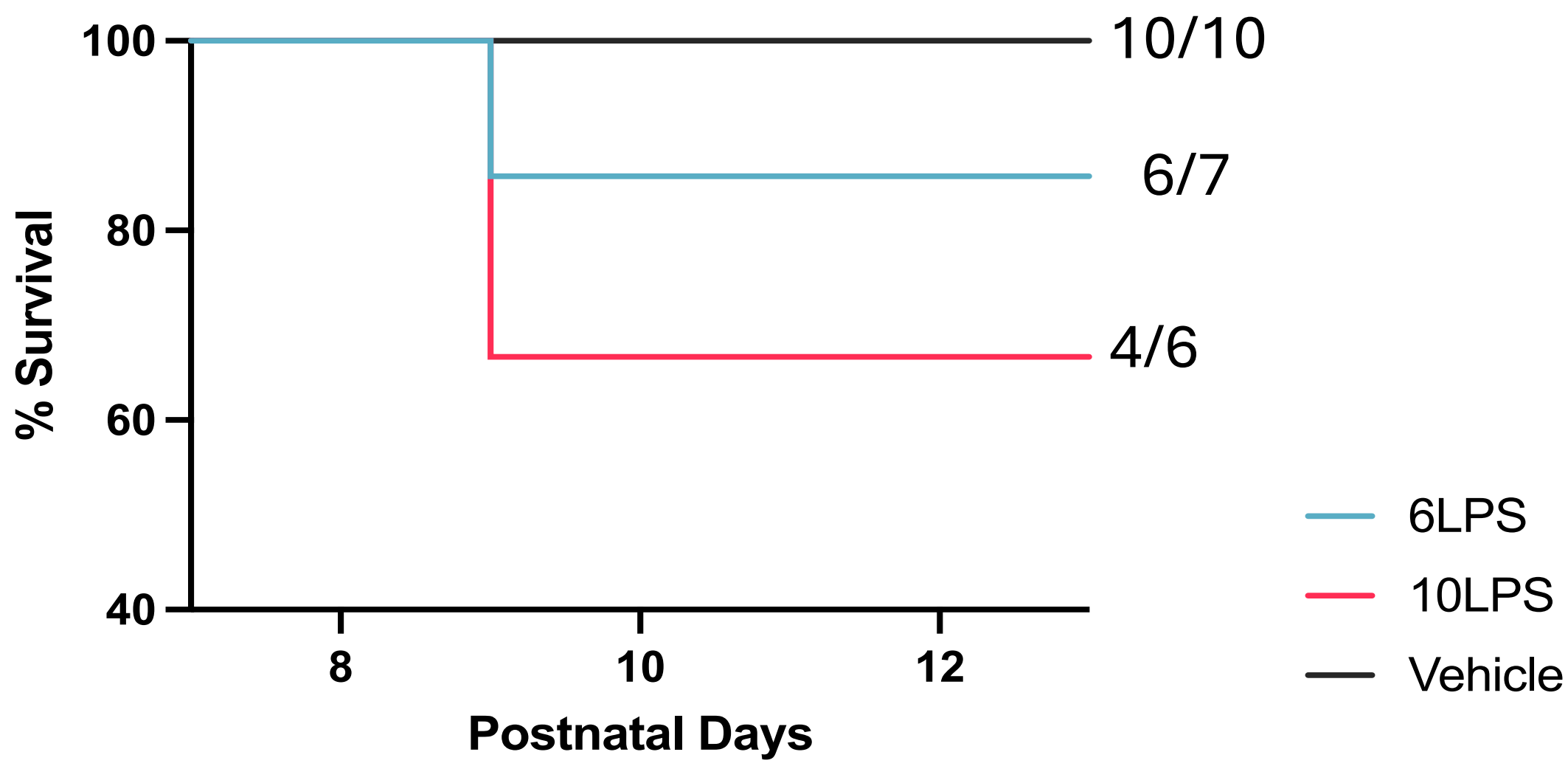

Figure S11. Kaplan-Meier survival curves. Survival rates in C57BL6 mice treated twice i.p. with LPS on P7-8. Blue line indicates the survival rate in mice treated twice with 6 mg/kg of LPS. Red line indicates the survival rate in mice treated twice with 10 mg/kg of LPS. Black line shows vehicle-treated controls. Data indicate that repeated exposure to increasing doses of LPS causes low probability of survival 24 hrs after the second LPS treatment (n=6-10/group).

Figure S12

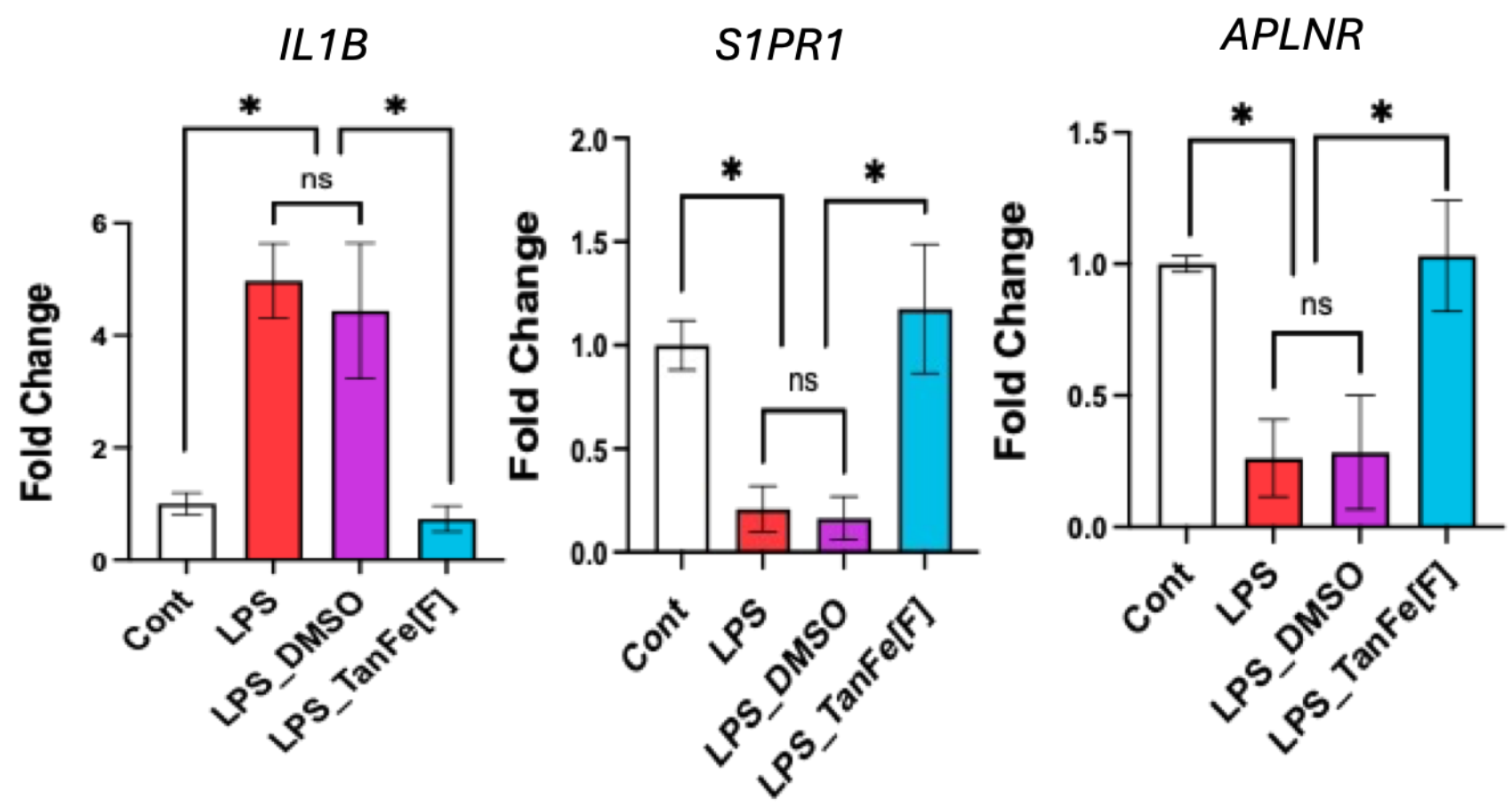

Figure S12: TanFe[F] influences mRNA levels of FOXF1 target genes in lung endothelial cell in vitro. Human pulmonary microvascular endothelial cells (HPMEC) were treated with 1 µg/mL LPS for 24 hrs, followed by exposure to 20 µM TanFe[F] or vehicle control for an additional 24 hrs. Cells were harvested 24 hrs post TanFe[F] treatment for gene expression analysis by qRT-PCR**.** Data indicate TanFe[F] reversed the LPS-induced induction of *IL1β* mRNA and restored *S1PR1* and *APLNR* mRNAs. Statistical significance was calculated using one-way ANOVA with Tukey's posthoc multiple comparisons. Data show mean ± SD, n=6-8/group. P≤0.05 is *, ns is not significant.

Figure S13

A. FOXF1$^+$ ECs-Split Channels (Control)

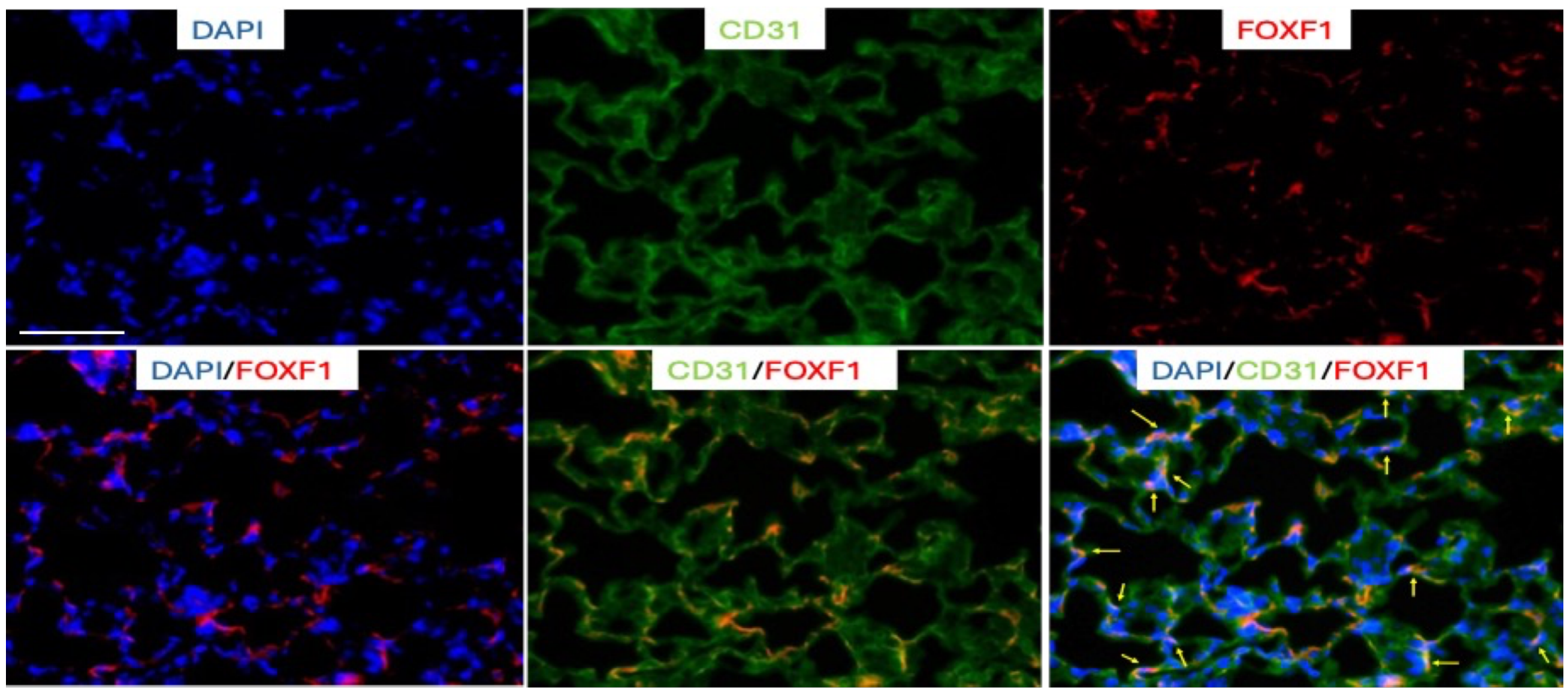

B. Ki67$^+$ ECs (Merged Channels)

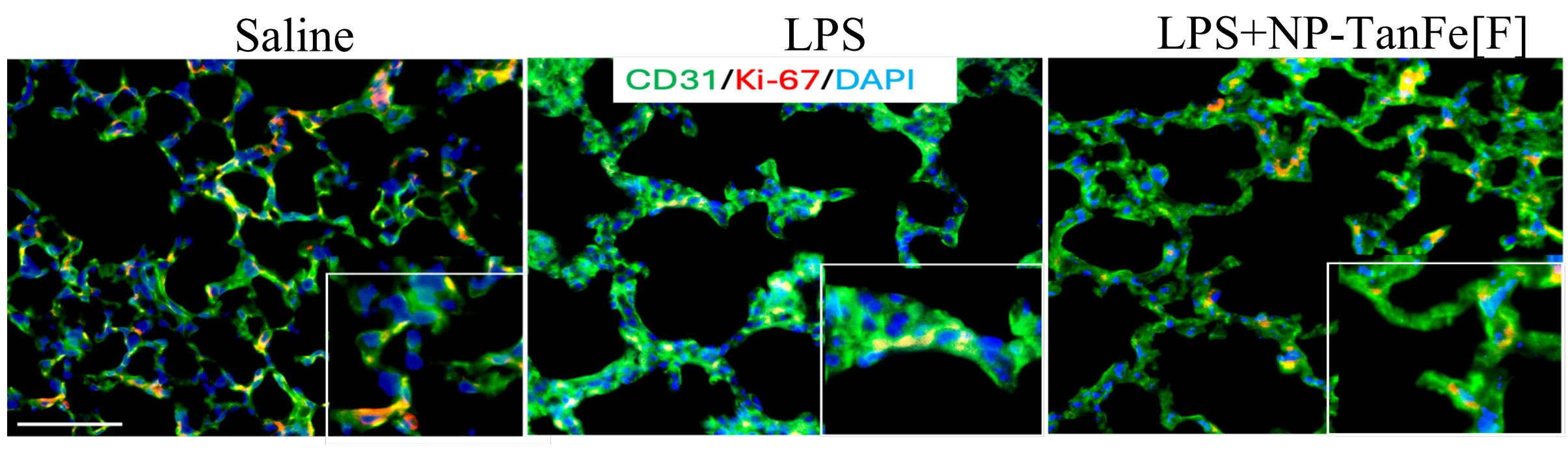

C. Ki67$^+$ ECs Split Channels (Control)

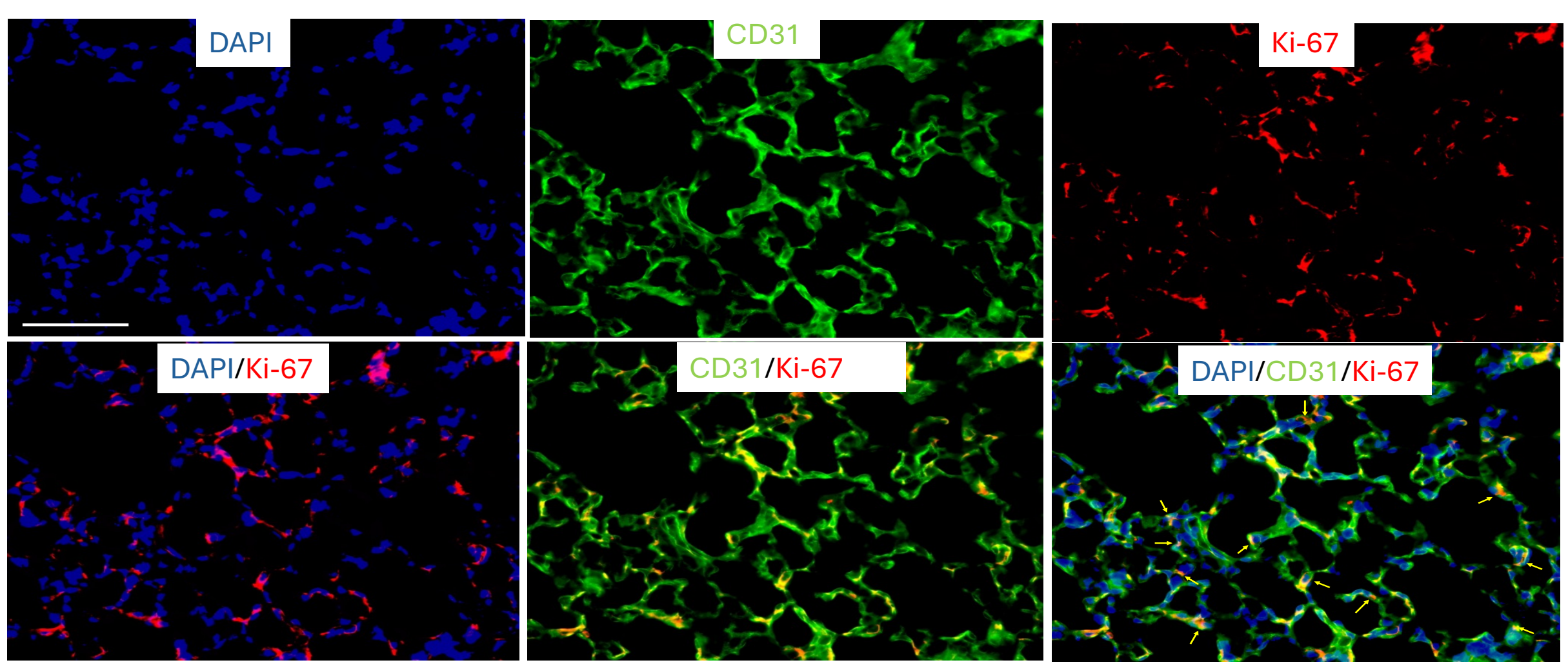

Figure S13. Immunostaining of lung frozen sections for FOXF1, Ki-67, and endothelial cell marker CD31. Mice were injured by LPS on P7 and P8, and treated with PBAE-1.4DA-PEG4X nanoparticles containing 0.4 μg of TanFe[F] on P9 and P10. Mice were harvested on P14. Lung sections were stained with antibodies against **(A)** CD31 and FOXF1 **(B-C)** CD31 and Ki-67. Scale bar is 50 microns. DAPI was used to quantify cell nulei (blue).

.

Figure S14

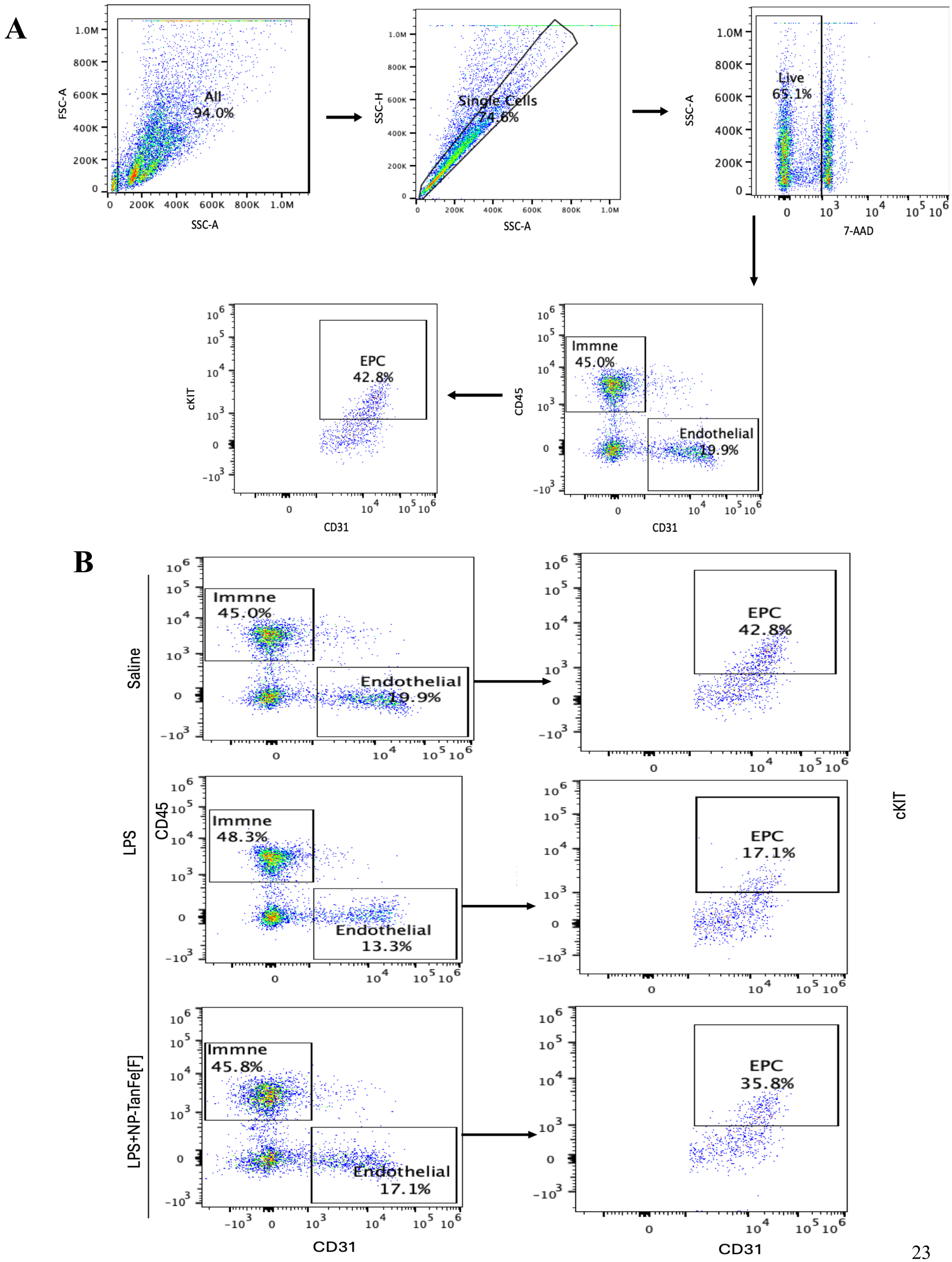
A
All
94.0%
FSC-A
SSC-A
Single Cells
74.6%
SSC-H
SSC-A
Live
65.1%
SSC-A
7-AAD
EPC
42.8%
cKIT
CD31
Immne
45.0%
Endothelial
19.9%
CD45
CD31
B
Saline
Immne
45.0%
Endothelial
19.9%
EPC
42.8%
LPS
CD45
Immne
48.3%
Endothelial
13.3%
EPC
17.1%
cKIT
LPS+NP-TanFe[F]
Immne
45.8%
Endothelial
17.1%
EPC
35.8%
CD31
CD31

Figure S14. Flow cytometry analysis was performed to identify endothelial cells, endothelial progenitor cells, and hematopoietic cell types. (A) Representative gating strategy to identify hematopoietic, endothelial cells (ECs), and endothelial progenitor cells (EPCs) in control uninjured mouse lungs. **(B)** Flow cytometry identifies pulmonary cell types in LPS-injured mice with or without nano-TanFe[F] treatment. LPS-induced decline in ECs and EPCs was improved in nano-TanFe[F] treated lungs.

Figure S15

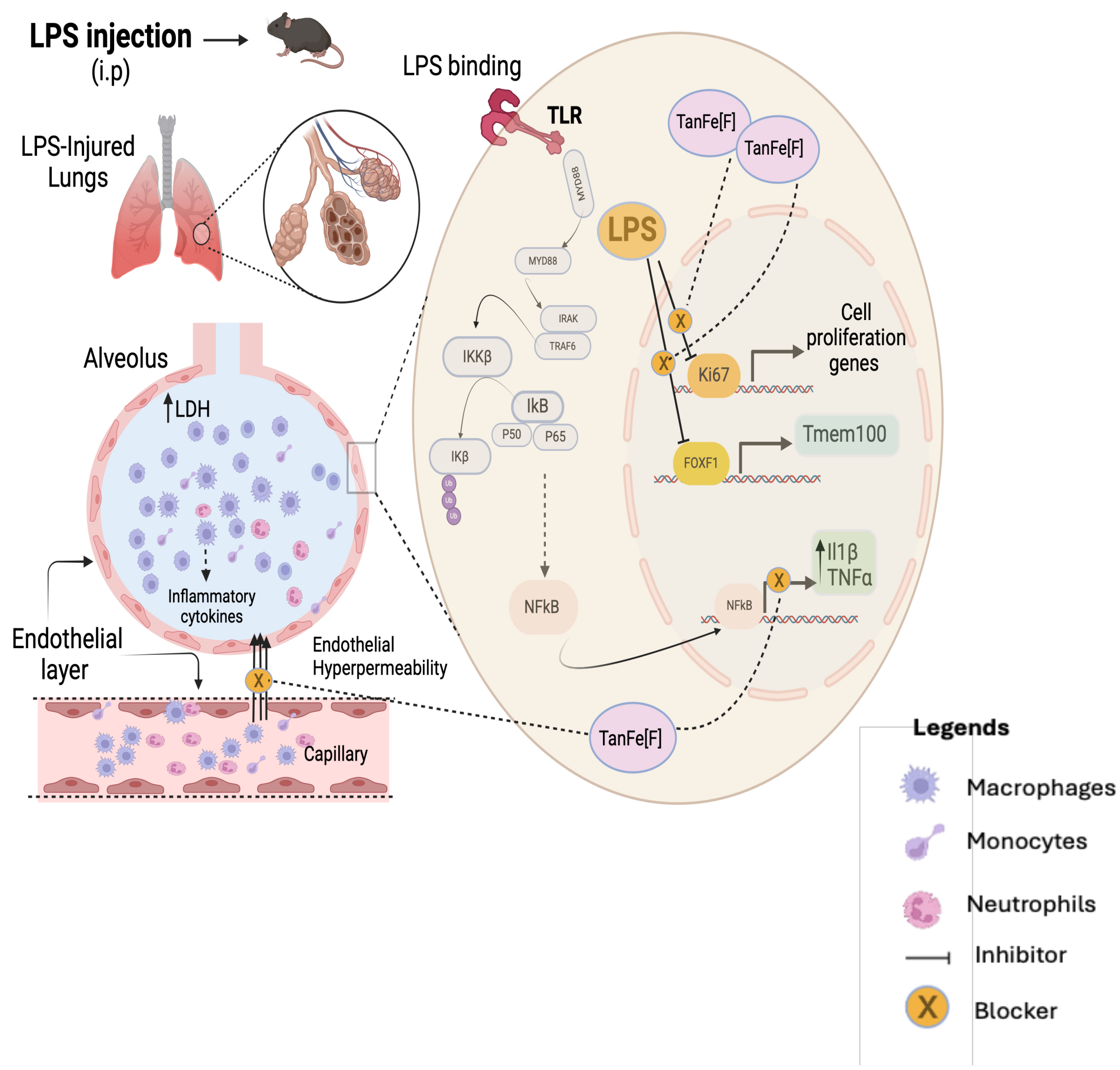

Figure S15. Graphical illustration of protective roles of TanFe[F] in mouse model of pediatric ARDS. Illustration shows molecular basis of LPS-induced injuries and beneficial effects of nano-TanFe[F] treatments in ARDS model. TanFe[F] increases FOXF1 protein in lung ECs, resulting in increased expression of proangiogenic EC genes and suppression of proinflammatory *Il1β* and *Tnfα*. TanFe[F] decreases LPS-induced EC permeability, increases EC proliferation, and decreases lung inflammation after LPS injury.
